# Comparing the tractability of young hand-raised wolves (*Canis lupus*) and dogs (*Canis familiaris*)

**DOI:** 10.1038/s41598-020-71687-3

**Published:** 2020-09-07

**Authors:** Dorottya Júlia Ujfalussy, Zsófia Virányi, Márta Gácsi, Tamás Faragó, Ákos Pogány, Boróka Mária Bereczky, Ádám Miklósi, Enikő Kubinyi

**Affiliations:** 1 MTA-ELTE Comparative Ethology Research Group, Budapest, Hungary; 2 Department of Ethology, ELTE Eötvös Loránd University, Pázmány Péter sétány 1/C, Budapest, 1117 Hungary; 3 Comparative Cognition, Messerli Research Institute, University of Veterinary Medicine, Vienna, Austria; 4 Medical University of Vienna, University of Vienna, Vienna, Austria; 5 Wolf Science Center, Domestication Lab, Konrad Lorenz Institute of Ethology, University of Veterinary Medicine Vienna, Vienna, Austria

**Keywords:** Developmental biology, Neuroscience, Zoology

## Abstract

Dogs live in 45% of households, integrated into various human groups in various societies. This is certainly not true for wolves. We suggest that dogs’ increased tractability (meant as individual dogs being easier to control, handle and direct by humans, in contrast to trainability defined as performance increase due to training) makes a crucial contribution to this fundamental difference. In this study, we assessed the development of tractability in hand-raised wolves and similarly raised dogs. We combined a variety of behavioural tests: fetching, calling, obeying a sit signal, hair brushing and walking in a muzzle. Wolf (N = 16) and dog (N = 11) pups were tested repeatedly, between the ages of 3–24 weeks. In addition to hand-raised wolves and dogs, we also tested mother-raised family dogs (N = 12) for fetching and calling. Our results show that despite intensive socialization, wolves remained less tractable than dogs, especially in contexts involving access to a resource. Dogs’ tractability appeared to be less context dependent, as they followed human initiation of action in more contexts than wolves. We found no evidence that different rearing conditions (i.e. intensive socialization vs. mother rearing) would affect tractability in dogs. This suggests that during domestication dogs might have been selected for increased tractability, although based on the current data we cannot exclude that the differential speed of development of dogs and wolves or the earlier relocation of wolves to live as a group explains some of the differences we found.

## Introduction

Due to domestication, behaviourally and morphologically, dogs (*Canis familiaris*) have greatly diverged from wolves (*Canis lupus*), even if they still crossbreed in nature and they are capable to produce fertile offspring, indicating an intermediate stage of speciation^1–4^. Albeit it is still strongly debated how dog behaviour has been altered by domestication, it remains to be an obvious difference that messes of only dogs can live, work and spend leisure time with humans safely and contently^5^. In contrast, keeping wolves in human homes typically imposes risks on both humans and wolves and compromises their well-being^6^. Early comparisons of human-raised wolves and dogs have suggested that the trainability (i.e*. the capacity of learning to perform behaviours upon command or context or in other words, increasing performance due to specific training*) and tractability (i.e. *controllability, handleability, manageability, adaptability to different contexts, accepting limitations and human guiding*) of dogs that have increased during domestication make a crucial contribution to this difference^7–9^. Frank^10^ found that dog puppies outperformed wolf pups in two training tasks where they either had to stay on a platform for 15 to 120 s or they had to walk on a leash next to their trainer with no tension on the leash. He also suggested that this difference is due to the increased tractability of dogs that he defined as “(a) responsiveness to a broad band of stimuli, including the sorts of verbal cues, mechanical manipulations, and reinforcers that humans use to communicate with preverbal children and animals, and (b) sufficient behavioural plasticity to permit desired responses to be externally shaped, after which the frequency of such responses is increased by reinforcement”^10^ (p. 144). In practice, tractability is the capability of being easily led, controlled, or easily handled, managed without showing resistance or objecting in other ways^11^. Difference in tractability between adult dogs and wolves has been shown recently in a cooperative problem solving task, as dogs followed their human partners more often and challenged their choices less frequently than wolves did^12^.

Less is known about dog-wolf differences in terms of trainability that seems to cover a partly overlapping range of skills with tractability (for exceptions see^13,14^). As within dogs, there seems to be a considerable across-individual variation in this trait, a substantial body of dog personality questionnaires has devoted several questions to assessing trainability (see^15^). For example, the Canine Behavioural Assessment and Research Questionnaire/C-BARQ^16^ uses eight questions to assess this trait in an individual (to be scored from 0–4) (1) when off leash, returns immediately when called; (2) obeys the sit command immediately; (3) obeys the stay command immediately; (4) seems to attend/listen closely to everything you say or do; (5) fast to respond to correction or punishment; (6) fast to learn new tricks or tasks; (7) difficult to distract by interesting sights, sounds or smells; (8) will ‘fetch’ or attempt to fetch sticks, balls, or objects. Performing well in these tasks requires skills in addition to tractability, such as low distractibility and attention to human actions. Informative in regard to possible effects of domestication is that owners report dog breeds that are genetically closer to wolves^17^) to be less trainable than less “wolf-like” breeds^18–20^. Furthermore, another study has compared the trainability of modern and basal dog breeds and domestic dingoes (*Canis dingo*) using the C-BARQ^21^. They found that dingoes were significantly less trainable than both basal and modern breeds, which may have resulted from the relatively recent^22^ lift of selection pressures of human environment on these dogs. These studies suggest that domestication has considerably affected the trainability of dogs. We suspect, however, that these differences in trainability in its conventionally broad sense (see 8 C-BARQ questions above), reflect differences in the animals’ tractability rather than other skills contributing to their trainability, and suggest that selection during the early stages of domestication affected primarily the animals’ tractability rather than their other skills, such as attentiveness for instance (see^23^ for a review on the similar social attentiveness of dogs and wolves).

In this study, we aimed at assessing tractability and its ontogeny in hand-raised wolves and similarly raised dogs using five different experimental tests: retrieving an object, calling, sitting on request, allowing fur to be brushed and walking in a muzzle. These are basic tasks and activities for an average family dog, usually not particularly challenging to learn or to perform, however, a certain level of tractability is necessary for all of them. As these tasks require the animals to comply with and follow human actions, and either tolerate some physical restriction or give access to a target object, we expected that dogs would show higher tractability than wolves. Additionally, we investigated environmental and developmental effects on tractability by comparing mother-raised and hand-raised dogs with intensive socialization in some of the cases and by testing the subjects repeatedly between the age of 6 and 24 weeks. We did not expect a difference in tractability between hand-raised and mother-raised dogs, as we suggest that a higher level of tractability in the case of dogs is a product of domestication and does not require intensive socialization to emerge relatively early in development^11^.

## Summary of results

We found that at 9 weeks of age hand-raised dogs chased and grabbed a target (paper ball) more often than hand-raised wolves. Dogs also retrieved the object more often than wolves (hw–hd: p < 0.001; hw–md: p < 0.01) who, if they had grabbed the ball, tended to carry it away (hw–hd: p = 0.11; hw–md: p = 0.03). Furthermore, unlike any of the dogs, 4 out of 16 wolves showed aggressive behaviour when the Experimenter tried to get the target object back. In contrast to fetching, hand-raised dogs and wolves behaved largely similarly when being called or requested to sit down for a piece of food. Only at older ages (16 and 24 weeks) and when being called in a social context, were wolves more difficult to call back than dogs (16 weeks: p = 0.04; 24 weeks: p = 0.02). Dogs were also quicker to sit for a reward than wolves at 7 (p < 0.01) and 16 weeks (p = 0.03) but not at 9, 12 and 24 weeks. When being brushed, wolves made more biting attempts than dogs at their age of 12 weeks (p = 0.01), however, this difference diminished by the age of 16 weeks when dogs also bit more often. Dogs and wolves also similarly accepted when getting a muzzle, still, dogs walked more readily with a muzzle on than wolves (p = 0.04), even if 24-week-old wolves performed at least as well as 16-week-old dogs. For a summary of our main results please see Table 1. All recorded data are supplied in Supplementary Information 2.

**Table 1:** Summary of tests, age of subjects during testing in weeks, maximum [Number of subjects (N) may differ between weeks (see details in the text)] number of subjects (hw: hand-raised wolves, hd: hand-raised dogs, md: mother-raised dogs), names and definitions of behavioural variables.

Test	Week	Hw N	hd N	md N	Variable name	Variable definition	Results
1. Fetching	6, 9	16	11	12	Retrieving (score 0–3)	Number of times (out of three) when the pup moved within 30 cm close to the experimenter while holding the paper ball in its mouth	Hw retrieved the ball fewer times than hd and md (Fig. 2A) All groups retrieved the ball more times at the age of 9 than 6 weeks
					Aggressive behaviour (score 0–3)	Number of times (out of three) when the pup produced any sign of aggression (growling, biting, snapping, snarling) while the experimenter was trying to take the ball away	4 out of 16 hw showed aggression while none of the hd and md (Fig. 2B)
					Carry away (score 0–3)	Number of times (out of three) when the pup moved away from the experimenter while holding the ball in its mouth	Hw carried away the ball more times than md (Fig. 2C) All groups carried away the ball more times at the age of 9 than 6 weeks
2. Calling	3, 4, 5, 6, 7, 8, 12, 16, 24	13	11	12	Latency to approach (sec)	Seconds from the start of the test until the subject approached the caregiver	Hw approached the caregivers slower than hd at the age of 16 and 24 weeks (Table 5)
3. Sitting on request	7, 9, 12, 16, 24	12	10	0	Latency to the first successful sitting (sec)	Seconds from the start of the test until the first time the subject sat down	Hw sat down slower than hd at the age of 7 and 16 weeks (Table 6)
4. Brushing	12, 16	11	10	0	Biting (n)	Number of biting attempts per episode	Hw showed more biting attempts than hd at the age of 12 weeks Hd showed more biting attempts at the age of 16 than 12 weeks (Fig. 5)
					Struggling (sec)	0—no struggling, to 3—continuous struggling	–
5. Muzzle	16, 24	13	10	0	Putting the muzzle on (score 0–3)	0—easy to put on, 3—very difficult to put on (due to the subject’s resistance)	–
					Walking with muzzle on (score 0–3)	0—not moving at all (standing still or struggling to get rid of muzzle), 3—walking undisturbed with muzzle on)	24-week-old dogs walked the most readily with muzzle on and 16-week-old wolves walked the least (Fig. 7)

## Discussion

In our five tests assessing various aspects of tractability and their changes during ontogeny, we found pronounced differences as well as similarities between hand-raised, intensively socialized wolves and dogs. In some tests, wolves’ performance was comparable to that of dogs, while in some other tests their tractability proved to be inferior. Among other aspects, we have tested how dogs and wolves *respond to physical restraint (brushing and wearing a muzzle)*, a characteristic that Gácsi et al.^11^ had reported to differ between dogs and wolves. In the social contexts applied in the present study, we found no clear differences between the two species in the brushing and muzzle tasks, as putting a muzzle on dogs and wolves was similarly easy, they struggled similarly long when being brushed and the difference in the number of their biting attempts diminished by their age of 24 weeks. We suggest that Gácsi et al.^11^ found larger differences because they measured the animals’ reaction to physical restraint while those wanted to search for food. In our brushing and muzzle tasks, the animals might have shown lower resistance because they were not restrained from accessing a resource. The importance of such a difference in context seems to be confirmed by the finding that dogs and wolves again clearly differed in the fetching task (e.g. wolves showing aggression and dogs not) where they likely perceived the paper ball as a resource. Accordingly, in this task, the wolves clearly showed more *avoidance* of the experimenter than the mother-raised dogs and more *aggression* than both dog groups when the experimenter tried to take the ball away from them.

Furthermore, the fetching task has been suggested to test another important component of tractability: *cooperativeness* in the sense of responding positively to human initiations of joint actions^24^. In this respect, we have found that even though a few of the wolves indeed repeatedly retrieved the paper ball upon being called by the experimenter, they did so significantly less often than either hand-raised or mother-raised dogs. Importantly, this difference does not seem to reflect a difference simply in the *trainability* of dogs and wolves, as we found no such profound differences between the two species in the sitting and calling tasks. It is especially relevant that at the ages of being tested in the fetching task, the wolves responded to being called as fast as the dogs, and a difference in this respect started to emerge only two months later in their development (both in the calling and in the muzzle tests). Therefore, we suggest that the difference in dogs’ and wolves’ fetching performance reflects their different ways of cooperating with humans (again, in the sense of responding positively to human initiations) that have been demonstrated also in a cooperative string-pulling task where adult animals could work together with humans to get access to food^12^. In both tasks, wolves acted more independently of their human partner’s actions as compared to dogs that followed their human partner’s initiations more readily.

In sum, our results suggest that dogs and wolves respond differently to physical restraint and inhibition primarily when thereby they are prevented from access to a resource (but see also^10^). The actual context may also, in the same way, influence how cooperative wolves are in following human invitations to interact and act together. Dogs appear to be less context dependent, more prepared to follow human initiation of action in most contexts. This may explain why the wolves were less cooperative and more aggressive in the fetching task and at the same time as tractable as the dogs in the calling and sitting tasks. These findings are in line with earlier studies that showed that hand-raised wolves at different ages can be as attentive and responsive to humans as dogs^25–29^, although dogs do not need specific and intensive socialization to acquire these abilities^11^.

It is also notable that in tests in which mother-raised dogs were also included (fetching and calling in at 6 and 8 weeks) we found no differences between them and the intensively socialized hand-raised dogs. This finding suggests that intensive socialization, or the lack of it, has no significant effect on dogs’ behaviour in these tasks at young ages. It is still possible, however, that early and intensive socialization either masked or enlarged genetically based differences between dogs and wolves. If equipped with different genetic predispositions and learning preferences, wolves and dogs, even if raised under identical conditions, likely gain different experiences, go through different learning processes, and, ultimately, adapt their behaviour to different aspects of the same environment^5,30^. Therefore, even if comparing animals raised in the same way, we can detect epigenetic differences between wolves and dogs that are likely to be more profound the older the investigated animals are^11,31^. The advantage of the current study is that we compared pups at young ages, thus the differences and similarities detected here had not yet been modified by developmental processes as strongly as in adult animals. However, comparing animals at such young ages along their chronological age carries the risk of detecting false differences if dogs and wolves develop at a different pace^5^. The higher number of biting attempts of wolves in the brushing task may indeed be such a difference, as dogs became as aggressive as wolves by their age of 24 weeks, even if they were less aggressive at the earlier test (see also^11^ about shifts in the development of canine social skills). Also, one may argue that testing dogs’ and wolves’ tractability at a relatively young age is unideal, as differences in tractability may become more pronounced with age.

Other limitations of the study are that our sample size was relatively low and because of the different length of hand-rearing, in tests conducted at the age of 12, 16, and 24 weeks, the majority of hand raised wolves had different experiences, which might have affected the results of the calling, sitting, brushing and muzzle test but not the fetching test. However, the socialization of the wolves continued in the Animal Park, where the cubs lived in the yard of the owner’s house on the premises, together with dogs, having human contact several times daily. Also, caregivers visited and walked the animals at least twice a week for a whole day, therefore they were called, asked to sit down and muzzled regularly while none of the subjects, including dogs were brushed before the tests. The pattern of the results suggests that the relocation of some of the wolves did not affect the results. For example, 2 “Sitting on command” tests were conducted before the relocation and the groups differed in one at the age of 7 weeks. 3 tests were conducted after the relocation and the groups differed only in one (at the age of 16 weeks). Another limitation is the fact, that while hand raised dogs were mixed breed of unknown genetic background, derived from village dogs, the mother raised dog group consists of various pure breeds, each selected for a certain purpose. We can unfortunately not rule out the possible effect of genetic background on our results, however the breed composition of the mother raised dog group is quite diverse, and genetic material of the Hungarian breeds included, such as the Pumi and the Puli, may also be found in the typical village mixed breed in Hungary.

Test conditions in some instances (e.g. calling in) varied during development and our results are based on single occasions, so we cannot infer to stability/consistency over time. Also, as already mentioned before, most of these test situations were not competitive, while our results on aggression and carrying an object away in the “fetching” test suggest that differences in such situations could be more pronounced.

It is also important to note that comparing today wolves to dogs has a limited potential to inform us about domestication. Wolves have likely faced selection by humans ever since domestication started. As such, since they have likely been selected for increased fear and avoidance of humans, the differences we find in the tractability of today wolves and dogs may overestimate the effects of domestication. Additionally, based on such behavioural comparisons it is impossible to tell whether the dog-wolf differences detected here originate from direct selection on these traits or from fundamental differences in dogs’ and wolves’ temperament. As such, dogs may turn out to be more tractable than wolves because they are more attracted to humans socially than wolves, or because they have a tamer temperament and/or reduced stress reactivity, as compared to wolves. Dog-wolf differences in the genetic variability of the oxytocin receptor gene and/or genes associated with hyper-sociability may contribute to the behavioural differences we found^32–34^.

Finally, it is important to note that, as long as comparative studies on aggressive behaviour and inhibition are still lacking, scarce or ambiguous^35^, we should take caution when interpreting similarities in wolf and dog behaviours. Not taking results on aggression into consideration can be very dangerous to humans and animals alike. However, socialization and training are valuable means of enrichment as well as a useful way to enhance husbandry related welfare for some captive animals^13,29^.

## General methods

### Subjects

Sixteen hand-raised grey wolf cubs (*Canis lupus*) and eleven similarly raised dog puppies (*Canis familiaris*) participated in our set of experiments, in addition to twelve mother-raised pet dog puppies in Test 1 and certain parts of Test 2. The wolves were born at Horatius Ltd. Animal Park (now Horkai Animal Training Centre, Hungary) in 2001, 2002, and 2004 in six litters. Hand-raised dogs (all mixed breed, breed composition unknown, derived from village dogs) were born at various animal shelters in Hungary, in 2000 and 2003 in five litters, while mother-raised pet dog subjects of different pure breeds were tested at their breeders’ homes in 2003 and 2004. All subjects were part of the wolf-dog comparative studies of the Family Dog Project (Department of Ethology, ELTE Budapest) and participated in some other experiments, results of which are already published^11,36–39^. More details on the subjects are given in Table 2. More details of the testing schedule and individual participation may be found in Supplementary Information 1.

**Table 2:** Details of subjects participating in the experiments (A, B, C, D, E, F are the litter identifiers in the three groups, M = male, F = female).

Name	Group	Born and tested	Litter	Sex
Barnus	Hand-raised wolf	2001	A	M
Rebeka	Hand-raised wolf	2001	A	F
Jimmy-Joe	Hand-raised wolf	2001	A	M
Minka	Hand-raised wolf	2001	A	F
Bence	Hand-raised wolf	2002	B	M
Bogi	Hand-raised wolf	2002	C	F
Zed	Hand-raised wolf	2002	B	M
Ursula	Hand-raised wolf	2002	D	F
Tóbiás	Hand-raised wolf	2002	B	M
Maja	Hand-raised wolf	2002	B	F
Zazi	Hand-raised wolf	2002	E	F
Léna	Hand-raised wolf	2002	E	F
Borisz	Hand-raised wolf	2002	C	M
Bodza	Hand-raised wolf	2004	F	F
Dakota	Hand-raised wolf	2004	F	M
Wolkó	Hand-raised wolf	2004	F	M
Boróka	Hand-raised dog	2000	A	F
Zokni	Hand-raised dog	2000	A	F
Stuka	Hand-raised dog	2000	A	M
Tódor	Hand-raised dog	2003	B	M
Füli	Hand-raised dog	2003	C	M
Tücsök	Hand-raised dog	2003	C	M
Maugli	Hand-raised dog	2003	D	M
Oszkár	Hand-raised dog	2003	C	M
Szofi	Hand-raised dog	2003	E	F
Arwen	Hand-raised dog	2003	E	F
Dodi	Hand-raised dog	2003	D	F
Puli_narancs	Mother-raised dog	2001	A	M
Puli_barna	Mother-raised dog	2001	A	F
Pumi_mokusC	Mother-raised dog	2003	B	M
Pumi_mocoN	Mother-raised dog	2003	B	F
Collie_2Rachel	Mother-raised dog	2003	C	M
Collie_2Rubin	Mother-raised dog	2003	C	F
Collie_3Sultan	Mother-raised dog	2003	D	M
Collie_3Shadow	Mother-raised dog	2003	D	F
Czechoslovakian_1w	Mother-raised dog	2004	E	M
Czechoslovakian_1l	Mother-raised dog	2004	E	F
Groenendale_sötétkék4	Mother-raised dog	2004	F	M
Groenendale_vilkék5	Mother-raised dog	2004	F	F

### Socialization and rearing

Hand-raised puppies, both wolf and dog, were separated from their mothers and littermates at the age of 4–6 days, when their eyes were still closed and were individually assigned to caregivers. The method of rearing and socialization was similar to that used by Fentress^40^, with the only exception that socialization began before the opening of eyes, that is, at a much earlier age (see^41,42^). Pups received a very intensive and sensitive human care, spending 22–24 h a day in close contact with their assigned caretaker. They were carried in pouches accompanying their caregivers throughout their everyday activities, to school, to work, in the car, on public transport, etc., thus they were daily exposed to novel visual, auditory and olfactory stimuli, humans, animals, and objects. Pups also had the opportunity to meet and socialize with their (age and litter) mates 2–3 times weekly. Pups were initially solely bottle-fed until the age of 5–6 weeks when solid food was gradually introduced. The basic handling principle, both in case of wolf and dog pups, was to avoid competitive, dominating situations, or aggressive interactions with the animals; we intended to use a similar approach observed in wolf mothers and adult pack members under natural circumstances^43^. Four wolf pups at the age of 24 weeks, the other animals at the age of 8–10 weeks were relocated to live in a mixed species (wolf and dog) group with regular human contact in the yard around the house of the owner at the Animal Park where they were born. Hand-raisers visited the cubs at least for two full days/week. Most of our hand raised dog pups were taken on by their caretakers for their entire lives, while some were taken on by adoptive families approx. at the age of 8–10 weeks. Members of our mother raised group moved from their breeder to their owner at around the age of 9 weeks and had not participated in further tests. Because of the different length of hand-rearing, in 3 test series, conducted at the age of 12, 16, and 24 weeks, 12 out of 16 hand raised wolves had different experiences than 4 wolves and all hand-raised dogs.

Our research team was licensed by the Department of Nature Conservation, Ministry of Environmental Affairs (No. 3293/2001), as well as the Ethical Committee for Animal Experimentation of Eötvös University to hand-rear and socialize the subjects and to conduct this research. The exact ages at which the reported tests were undertaken will be reported in the methods section of each test.

### Ethical statement

The reported research project is in accordance with the Ethical Guidelines of Research in Hungary, has been conducted with permission from the Department of Nature Conservation, Ministry of Environmental Affairs (No. 3293/2001), as well as the Ethical Committee for Animal Experimentation of the Eötvös Loránd University of Sciences. Methods are also in agreement with ASAB guidelines.

### Informed consent to images

All identifiable persons (Zsófia Virányi—the second author, Dóra Újváry—former research associate, and Dorottya Júlia Ujfalussy—first author) gave informed consent to the publication of their pictures as part of this manuscript.

## Test 1: fetching (object retrieval)

### Methods

#### Subjects

All subjects shown in Table 2, that is, 16 hand-raised wolves (hw), 11 hand-raised dogs (hd) and 12 mother-raised dogs (md) were involved in this experiment.

#### Experimental procedure

Subjects were repeatedly tested at 6 and 9 weeks of age, in the same experimental tests.

Each subject was tested by a female experimenter, in absence of their caregiver, in one of two empty test rooms (floor dimensions: 3 × 4.5 m and 2.5 × 4 m) that the subjects were allowed to enter only during the tests. A female experimenter recorded the tests using a handheld camera.

**Figure 1:**
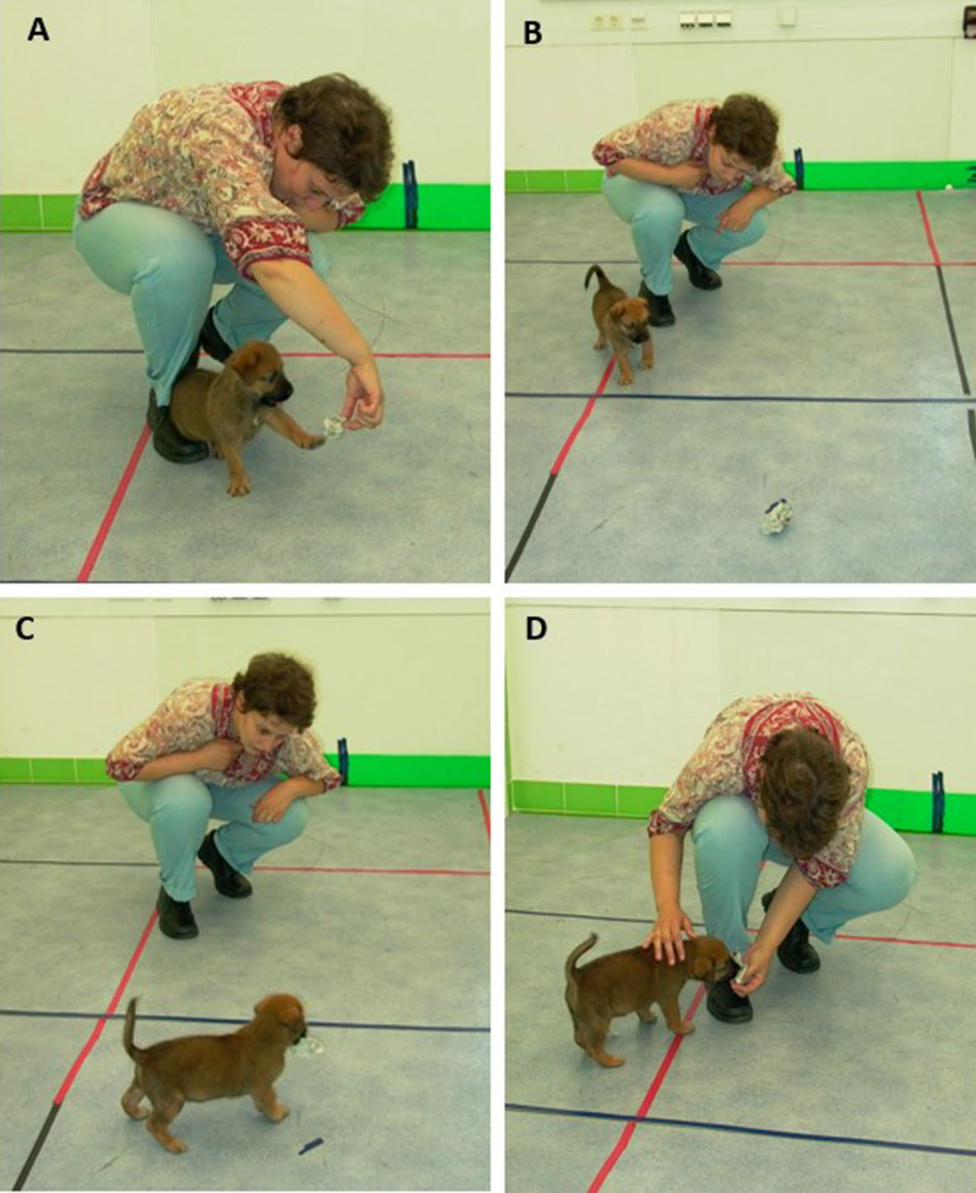
Fetching. The experimenter manipulated a paper ball playfully in front of the pup (**A**). When the pup was focusing on the object, she threw the ball approximately 50–100 cm away and waited silently for 15 s (**B**). If the pup went for the ball and grabbed it (**C**), she called it back (“Come, come here!”) for maximum 30 s and took the ball away from the pup (**D**).

At the start, the experimenter manipulated a paper ball playfully in front of the pup. When the pup was focusing on the object, she threw the ball approximately 50–100 cm away and waited silently for 15 s. If the pup went for the ball and grabbed it, she called it back (“Come, come here!”) for maximum 30 s and took the ball away from the pup. In 30 s she could take the ball away from every pup. The ball was thrown three times in a row (Fig. 1).

#### Behavioural measures and data analysis

Video recordings were behaviourally coded for retrieving, carrying, and aggressive behaviours in each episode. For definitions of these behaviours, see Table 3.

**Table 3:** Behavioural variables scored in the object retrieving experiment with their definitions.

Behavioural variable	Definition
Retrieving (score 0–3)	Number of times (out of three) when the pup moved at least 30 cm close to the experimenter while holding the paper ball in its mouth
Aggressive behaviour (score 0–3)	Number of times (out of three) when the pup produced any sign of aggression (growling, biting, snapping, snarling) while the experimenter was trying to take the ball away
Carry away (score 0–3)	Number of times (out of three) when the pup moved away from the experimenter while holding the ball in its mouth

#### Statistical analyses

Statistical analyses were carried out using R statistical environment (v. 3.4.2^44^. The proportion of the three trials when the subject responded by the pre-defined behaviour (see Table 2) were analysed in three separate binomial Generalized Linear Mixed Models (R package “lme4”^45^. Age (factor with 2 levels: 6 and 9 weeks of age), experimental group (factor with three levels: hand-raised dog, hand-raised wolf, and mother-raised dog) and their two-way interactions were included as fixed effects in initial models in addition to dog ID as a random term. Stepwise model selection was based on AIC values; the new model was considered better whenever delta AIC was above 2. We provide *χ*^2^ and *p*-values of likelihood ratio tests of models with and without the explanatory variable. Odds ratios are provided between levels of a given fixed effect. Post-hoc pairwise comparisons were performed using R package “lsmeans”^46^ applying Tukey correction. The final models are reported in the results.

### Results

#### Retrieving

**Figure 2:**
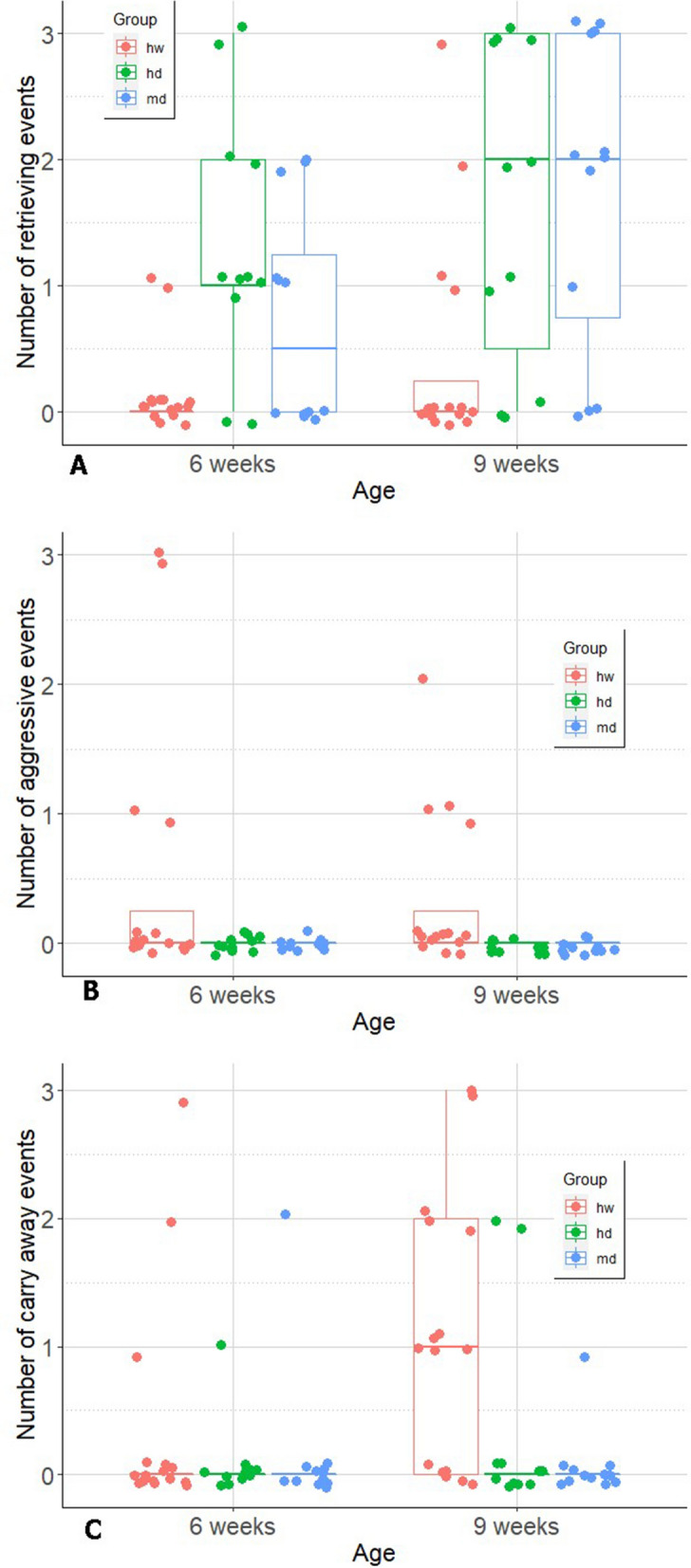
Line shows median, boxes the interquartile range, whiskers the range, while the dots represent the individual scores in retrieving the ball (**A**), showing aggression (**B**) or carrying the ball away (**C**) in 0, 1, 2 or 3 trials at the age of 6 and 9 weeks. hw = hand-raised wolves (N = 16), hd = hand-raised dogs (N = 11), md = mother-raised dogs (N = 12). Figure created with ggplot2^47^.

The final model of retrieving included the main effects of age and experimental group. Wolves, as well as the two groups of dogs, retrieved the ball in significantly more trials at week 9 than week 6 (odd.r (95% CI) 3.67(1.71–7.84); z = 3.35; p < 0.001). Pairwise comparisons showed that wolves retrieved the paper ball less often than both dog groups, while the latter two did not differ in this behaviour (hw-hd: odd.r (95% CI) 0.05 (0.01–0.23); z = − 3.78; p < 0.001, hw-md: odd.r (95% CI) 0.08 (0.02–0.35); z = − 3.31; p < 0.01, hd-md: odd.r (95% CI) 1.64 (0.40–6.76); z = 0.68; p = 0.78, Fig. 2A).

#### Aggressive behaviour

We observed aggressive behaviour only in wolves and it did not change with age (odd.r (95% CI) 0.48 (0.12–1.96); z = − 1.02; p = 0.31). Four of the 16 wolves showed aggressive behaviour when the Experimenter took the paper ball away. Two of these individuals were aggressive on all three occasions. None of the dogs on any of the occasions displayed aggressive behaviours in this situation (Fig. 2B).

#### Carry away

The final model of carrying away included also the main effects of age and experimental group. Older subjects carried away the paper ball significantly more than younger ones (odd.r (95% CI) 3.77(1.44–9.87); z = 2.70; p = 0.01). Difference between experimental groups was mainly driven by wolves that carried away the paper ball significantly more often than mother-raised dogs (hw-hd: odd.r (95% CI) 5.63 (1.06–29.87); z = 2.03; p = 0.11, hw-md: odd.r (95% CI) 11.71 (1.83–74.90); z = 2.60; p = 0.03, hd-md: odd.r (95% CI) 2.08 (0.27–16.01); z = 0.70; p = 0.76, Fig. 2C).

## Test 2: calling

### Methods

#### Subjects

Hand-raised wolves and hand-raised dogs were tested in this experiment at 3, 4, 5, 6, 7, 8, 12, 16 and 24 weeks of age. Mother-raised dogs were included only in the tests at 6 and 8 weeks of age. Thirteen hand-raised wolves, 11 hand-raised dogs, and 12 mother-raised dogs were involved in this experiment, however, groups and subject numbers varied between tests, due to occasional unavailability of subjects or caregivers.

#### Experimental procedure

The difficulty of the calling tests increased with ages to follow the development of subjects. Exact distances and conditions are shown in Table 4 below.

**Table 4:** Distances and conditions of the calling in test at different ages.

Age	Distance	Location	Context
3 week	1 m	Indoor	Alone
4 week	2 m	Indoor	Alone
5 week	5 m	Outdoor	Alone
6 week	5 m	Outdoor	Alone
7 week	5 m	Indoor	Alone
8 week	10 m	Outdoor	Alone
12 week	10 m	Outdoor	Paired
16 week	10 m	Outdoor	Paired
24 week	≈ 10 m	Outdoor	From playgroup

**Figure. 3:**
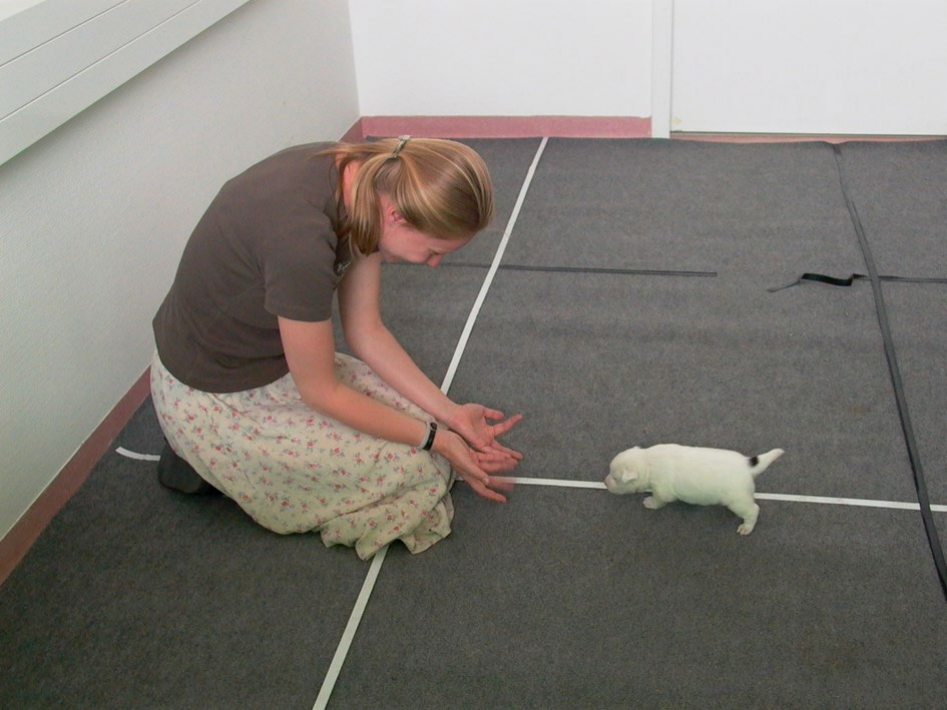
Calling at the age of 3 weeks.

Subjects were held by a familiar experimenter while their caretaker took a position at a given distance to them (see Table 4). After calling the subject’s attention by clapping her hands three times, the caretaker started to call the subject by their names and saying “come, come”. Once the caretaker has started to call the subject, the experimenter let it go. The caretaker kept calling the subject until it approached and physically contacted her, or until the test was terminated (Fig. 3). In the tests at weeks 12 and 16 the subjects were tested in pairs, that is, two individuals were called at the same time by their caregivers who were crouching next to each other (ca. 2 m distance from each other at the start). In the test at 24 weeks of age subjects were not held until calling started, but played in a playgroup with pack mates (Table 4). In all test occasions, calling was repeated twice in an identical manner and average latencies were calculated for the analysis.

#### Behavioural measures and data analysis

Latency to approach has been coded from the video recordings. Latency of approaching the caretaker was used as response variable in separate cox regression models for the different ages because the different experimental conditions and locations hindered direct developmental comparisons. In each model, experimental group was included as main effect. Latencies (seconds) to approaching the caretaker were analysed in Cox Regressions (R package “survival”) with occurrence of approaching as terminal event. Subjects that did not approach the caretaker were treated as censored observations.

### Results

We found a significant difference only between hand-raised dog and wolf puppies at 16 and 24 weeks of age. At both ages dogs approached the experimenter with lower latencies (quicker) than wolves (no group differences at any other ages; for statistical details, see Table 5.). Mean latencies and standard errors are given in Supplemetary Information 3. for possible comparisons.

**Table 5:** Results of the statistical analyses (Cox Mixed Models) at different ages.

Age	Contrast	Odds.r	95% CI	z	p	LogLik ratio	p	
3	hw–hd	0.48	0.17–1.34	− 1.40	0.16	2.04	0.15	
4	hw–hd	0.72	0.29–1.79	− 0.71	0.48	0.52	0.47	
5	hw–hd	1.47	0.63–3.39	0.89	0.37	0.79	0.38	
6	hw–hd	0.98	0.37–2.57	− 0.05	0.96	2.49	0.29	
	hw–md	0.50	0.19–1.32	− 1.41	0.16			
	hd–md	1.96	0.68–5.63	1.25	0.43			
7	hw–hd	1.69	0.60–4.75	0.99	0.32	0.98	0.32	
8	hw–hd	0.71	0.26–1.96	− 0.67	0.51	0.45	0.80	
	hw–md	0.86	0.34–2.20	− 0.31	0.76			
	hd–md	1.22	0.46–3.21	0.40	0.92			
12	hw–hd	2.03	0.86–4.82	1.60	0.11	2.55	0.11	
16	hw–hd	3.79	1.11–12.90	2.13	0.03	4.43	**0.04 (hw > hd)**	
24	hw–hd	4.49	1.12–16.94	2.22	0.03	5.94	**0.02 (hw > hd)**	

Significant differences are shown in bold.

## Test 3: sitting on command

### Methods

#### Subjects

12 hand-raised wolves and 10 hand-raised dogs were involved in this experiment at 7, 9, 12, 16 and 24 weeks of age.

#### Experimental procedure

**Figure 4:**
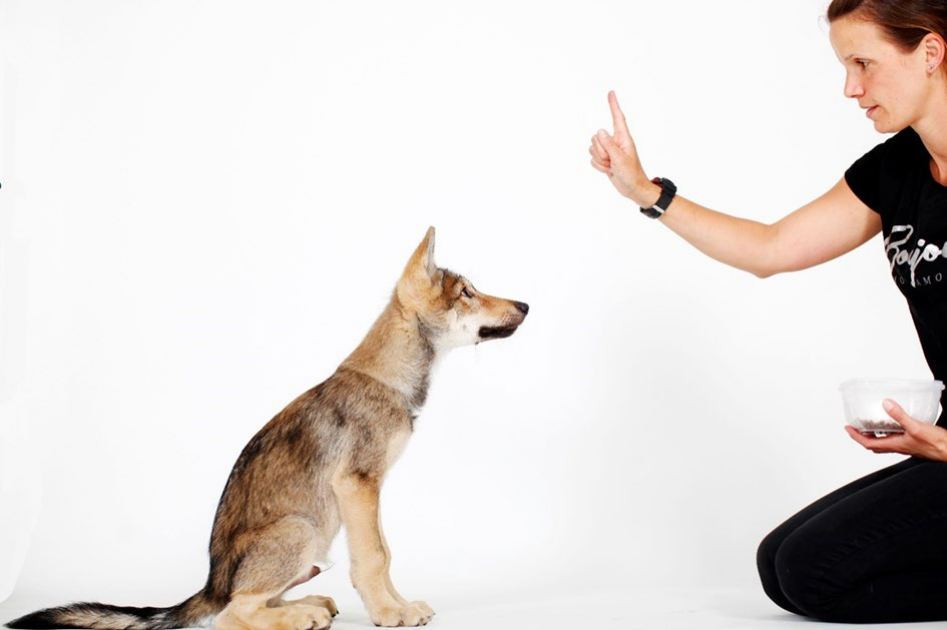
Sitting on command at the age of 16 weeks.

The experimenter held a piece of food reward approximately 30 cm above the nose of the standing subject, and moved it backward, thereby trying to get the subject to sit down and gave the verbal signal “sit”. If and when the subject eventually sat down, the reward was handed over. This process was continued until the subject first sat down upon instruction. In the first testing occasion, the animals had not yet been trained on the sit command, which however has then frequently been used during the everyday life of the animals as they were growing. Therefore, older animals were more familiar with and better trained on this command (Fig. 4).

#### Behavioural measures and data analysis

Latency to the first successful sitting has been coded from video recordings. Latency to first sitting was then analysed in Cox Mixed Models. Age, group and their two-way interaction were included as fixed effects, and subject ID as a random factor in the initial model.

### Results

The initial model already included a significant interaction between age and group (χ^2^ (4) = 12.62; p = 0.0133). The post-hoc pairwise comparisons revealed that the group difference was significant only at the age of 7 and 16 weeks. In both cases hand raised dogs performed the task faster (with lower latencies) than the wolves (Table 6). Mean latencies and standard errors are given in Supplemetary Information 3. for possible comparisons.

**Table 6:** Statistical details (expβ, standard error, z and p values) of the post-hoc pairwise comparisons of hand-raised wolves and dogs in the Sitting on command test.

Age	Odd.r	95% CI	z	p
7	0.13	0.04–0.45	− 3.29	**< 0.01 (hw > hd)**
9	1.30	0.45–3.78	0.48	0.63
12	1.26	0.53–3.01	0.54	0.59
16	0.31	0.12–0.87	− 2.23	**0.03 (hw > hd)**
24	0.73	0.29–1.88	− 0.65	0.52

Significant differences are shown in bold.

## Test 4: brushing

### Methods

#### Subjects

Subjects were tested in the brushing test at 12 and 16 weeks of age. At 12 weeks, 11 hand-raised wolves and 10 hand-raised dogs were involved, while at 16 weeks, 13 hand-raised wolves and 8 hand-raised dogs took part in this experiment.

#### Experimental procedure

In an indoor familiar experimental room, the subjects were placed on the floor, wearing a collar and a leash, held by a familiar experimenter. The experimenter attempted to brush the fur of the subjects continuously for 30 s with a dog grooming brush, covering all regions of the body except for the legs. The caregiver was present in the room but was passive throughout the testing.

#### Behavioural measures and data analysis

The number of biting attempts was counted and the overall amount of struggling (fidgeting) of the subjects during brushing was assessed (scores of moving: from 0—no struggling to 3—continuous struggling) from the video recordings.

Biting attempts were analysed using Generalised Linear Mixed Models with negative binomial distribution (R package “lme4”^45^, while struggling was analysed as an ordinal response variable in Mixed Effects Ordinal Regression Models (R package “ordinal”^48^. In both initial models, age, group and their two-way interaction were included as main effects. Stepwise model selection was applied based on AIC differences and likelihood ratio test.

### Results

#### Biting

**Figure 5:**
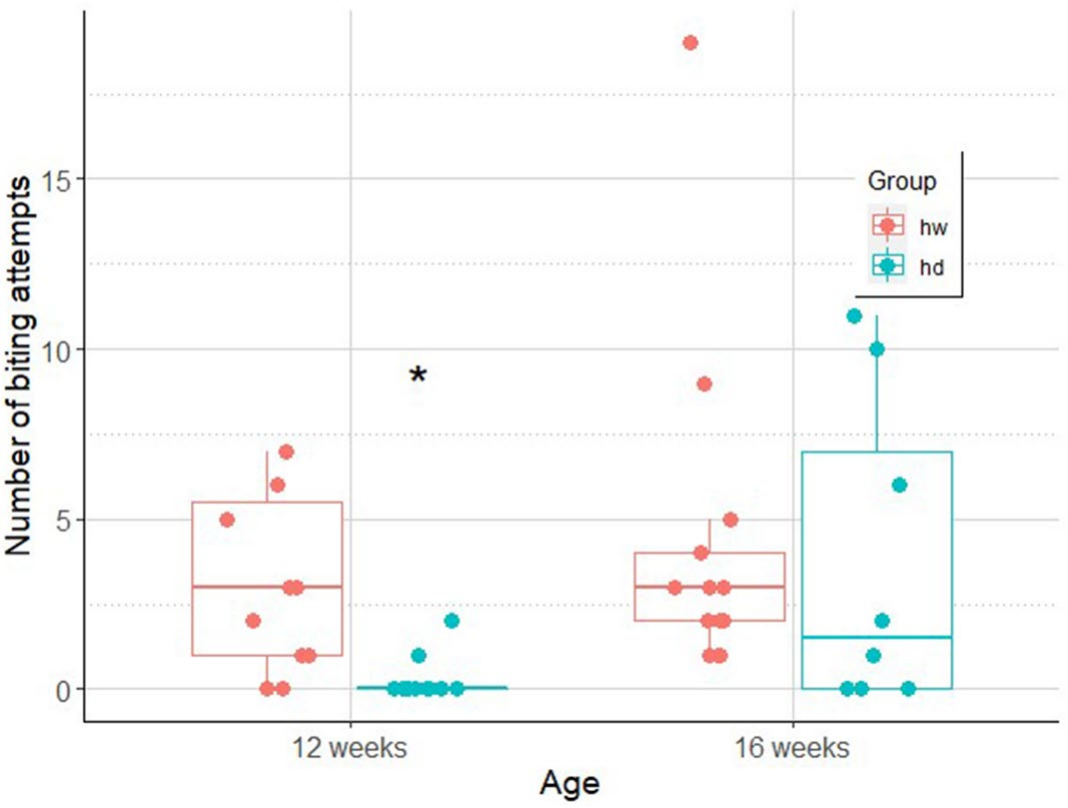
Number of biting attempts in hand–raised dog (hd) and hand-raised wolf (hw) groups at the age of 12 and 16 weeks (line: median, box: interquartile range, whisker: range, dots: actual data points of the individuals). Figure created with ggplot2^47^.

The final model included the interaction of age and group (odds.r. (95% CI) 9.47 (2.02–44.37); z = 2.86; p < 0.01). Dogs were less likely to attempt to bite when tested at 12 weeks of age, as compared to wolves (odds.r. (95% CI) 11.59 (2.74–48.94); z = 3.33; p = 0.01). The frequency of biting, however, has increased in dogs by the age of 16 weeks (odds.r. (95%CI) 0.08 (0.02–0.33); z = − 3.52; p < 0.01), when the difference between dogs and wolves diminished (Fig. 5).

#### Struggling

**Figure 6:**
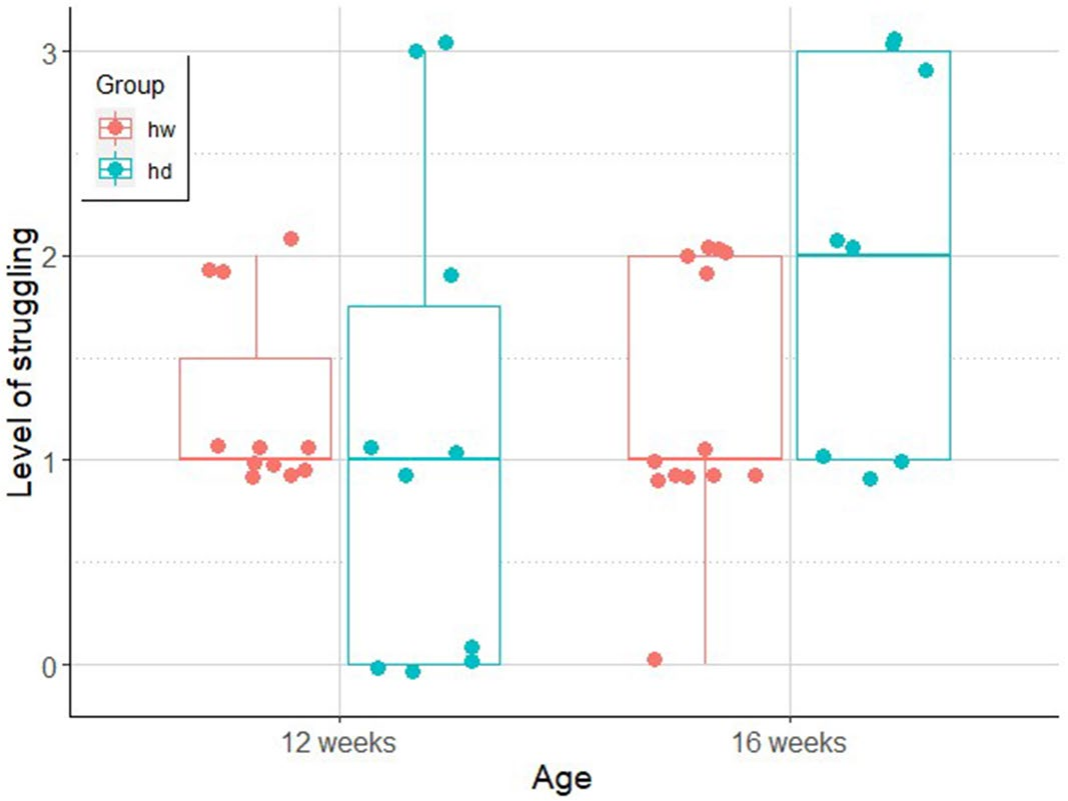
The level (0–3) of struggling (line: median, box: interquartile range, whisker: range, dots: actual data points of the individuals) while being brushed in our hand-raised dog (hd) and hand-raised wolf (hw) groups at the age of 12 and 16 weeks. Figure created with ggplot2^47^.

The final model of struggling included the interaction effect between age and experimental group, although it was not statistically significant (odds.r. (95% CI) 81.94 (0.73–9,242.43); z = 1.83; p = 0.07, Fig. 6).

## Test 5: muzzle and walking

### Methods

#### Subjects

Subjects were tested in the muzzle test at 16 and 24 weeks of age. At 16 weeks, 13 hand-raised wolves and 8 hand-raised dogs were involved, whereas, at 24 weeks, 8 hand-raised wolves and 10 hand-raised dogs took part in this experiment.

#### Experimental procedure

The caretaker put a standard wire and leather muzzle on the subject and subsequently started walking with the subject on the leash for 60 s. At this age, the animals were used to walking on the leash but had not been trained on wearing a muzzle.

#### Behavioural measures and data analysis

The difficulty of putting the muzzle on the animal, as well as the quality/undisturbed nature of the walking phase have been scored 0–3 *(putting the muzzle on*: 0—easy to put on, 3—very difficult to put on (due to the subject’s resistance); *walking with muzzle on*: 0—not moving at all (standing still or struggling to get rid of muzzle), 3—walking undisturbed with muzzle on). Struggling with the muzzle and walking were considered as ordinal response variables and were analysed in Mixed Effects Ordinal Regression Models (R package “ordinal”^48^. In both initial models, age, group and their interaction were included as main effects. Stepwise model selection was based on AIC differences.

### Results

#### Putting the muzzle on

None of the investigated variables, nor their interaction explained the difficulty to put the muzzle on (age: odds.r. (95% CI) 0.39 (0.04–4.05); z = − 0.79; p = 0.43; experimental group: odds.r. (95% CI) 0.75 (0.10–5.49); z = − 0.28; p = 0.78; Age-Group interaction: odds.r. (95% CI) = 4.20 (0.19–92.84); z = 0.91; p = 0.36).

#### Walking with muzzle on

**Figure 7:**
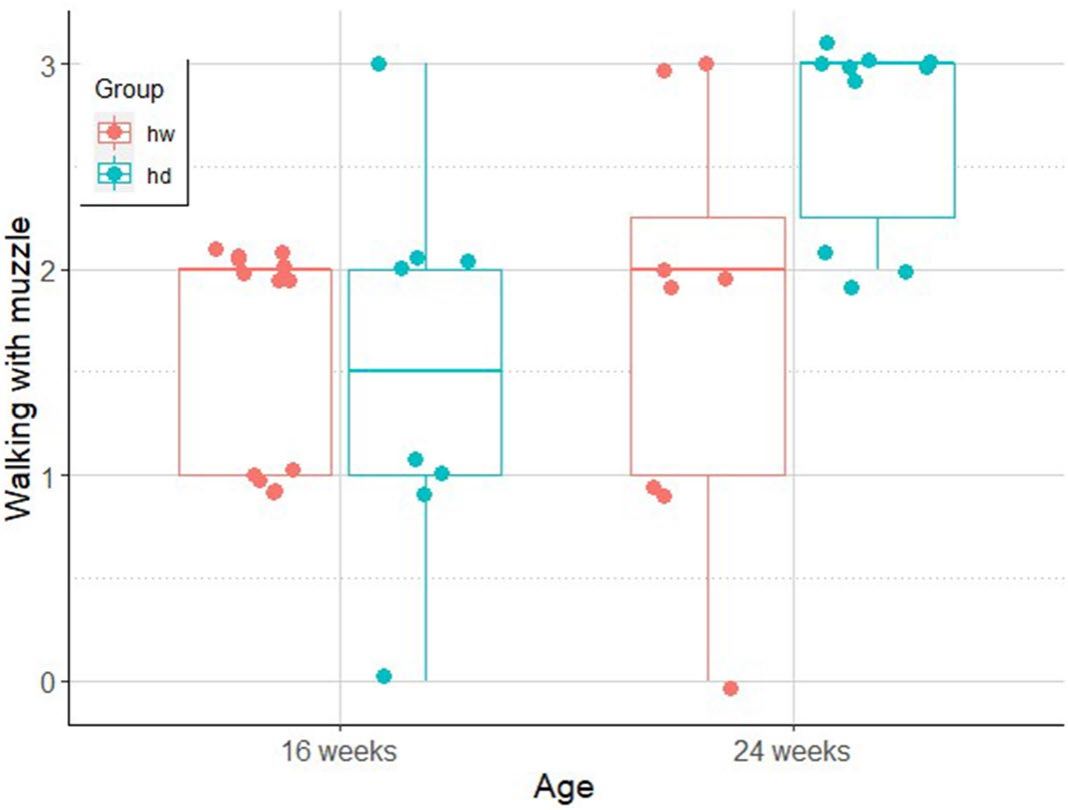
The degree (0–3) of moving along readily while wearing the muzzle in hand-raised dog (hd) and hand-raised wolf (hw) groups at the age of 16 and 24 weeks. (line: median, box: interquartile range, whisker: range, dots: actual data points of the individuals) Figure created with ggplot2^47^.

The final model included the significant interaction effect of age and group (odds.r. (95% CI) 34.33 (1.37–861.13); z = 2.15; p = 0.03) suggesting that 24-week-old dogs walked the most readily with muzzle on and 16-week-old wolves walked the least readily (odds.r. (95% CI) 0.01 (0.00–0.33); z = − 2.62; p = 0.04; Fig. 7).

## Supplementary information

Supplementary Information 1.

Supplementary Information 2.

Supplementary Information 3.

## Data Availability

All data generated or analysed during this study are included in this published article and its Supplementary Information files: SupplMaterial1ALLSUBJECTS.pdf, SupplMaterial2ALLDATA.pdf. and SupplMaterial3Test2and3latSE.pdf.
