# Supplementary material for: Comparing the tractability of young hand-raised wolves (Canis lupus) and dogs (Canis familiaris)
Source: Sci Rep. 2020 Sep 7;10:14678. doi: 10.1038/s41598-020-71687-3 (PMC7477132; doi:10.1038/s41598-020-71687-3)
Supplement: Supplementary file 1 — Supplementary Information 1. [file 41598_2020_71687_MOESM1_ESM.pdf]

Comparing the tractability of young hand-raised wolves (*Canis lupus*) and dogs (Canis familiaris)

Dorottya Júlia Ujfalussy [2*], Zsófia Virányi [3,4], Márta Gácsi [1,2], Tamás Faragó [2], Ákos Pogány 2, Boróka Mária Bereczky 1, Ádám Miklósi 2, Enikő Kubinyi 2

1 MTA-ELTE Comparative Ethology Research Group
2 Department of Ethology, ELTE Eötvös Loránd University, Budapest
3 Comparative Cognition, Messerli Research Institute,
University of Veterinary Medicine, Vienna, Medical University of Vienna, University of Vienna
4 Wolf Science Center, Domestication Lab, Konrad Lorenz Institute of Ethology,
University of Veterinary Medicine Vienna, Vienna, Austria

Supplementary material 1 - All subject data

Experiment	Name	Tested	Age	Group	Experiment	Name	Tested	Age	Group
Fetching	**Barnus**	2001.	6 w	hw	Calling in	**Barnus**	2001.	3 w	hw
Fetching	**Rebeka**	2001.	6 w	hw	Calling in	**Rebeka**	2001.	3 w	hw
Fetching	**Jimmy-Joe**	2001.	6 w	hw	Calling in	**Minka**	2001.	3 w	hw
Fetching	**Minka**	2001.	6 w	hw	Calling in	**Bence**	2002.	3 w	hw
Fetching	**Bence**	2002.	6 w	hw	Calling in	**Bogi**	2002.	3 w	hw
Fetching	**Bogi**	2002.	6 w	hw	Calling in	**Zed**	2002.	3 w	hw
Fetching	**Zed**	2002.	6 w	hw	Calling in	**Ursula**	2002.	3 w	hw
Fetching	**Ursula**	2002.	6 w	hw	Calling in	**Tóbiás**	2002.	3 w	hw
Fetching	**Tóbiás**	2002.	6 w	hw	Calling in	**Maja**	2002.	3 w	hw
Fetching	**Maja**	2002.	6 w	hw	Calling in	**Zazi**	2002.	3 w	hw
Fetching	**Zazi**	2002.	6 w	hw	Calling in	**Léna**	2002.	3 w	hw
Fetching	**Léna**	2002.	6 w	hw	Calling in	**Borisz**	2002.	3 w	hw
Fetching	**Borisz**	2002.	6 w	hw	Calling in	**Tódor**	2003.	3 w	hd
Fetching	**Bodza**	2004.	6 w	hw	Calling in	**Füli**	2003.	3 w	hd
Fetching	**Dakota**	2004.	6 w	hw	Calling in	**Tücsök**	2003.	3 w	hd
Fetching	**Wolkó**	2004.	6 w	hw	Calling in	**Maugli**	2003.	3 w	hd
Fetching	**Boróka**	2000.	6 w	hd	Calling in	**Oszkár**	2003.	3 w	hd
Fetching	**Zokni**	2000.	6 w	hd	Calling in	**Szofi**	2003.	3 w	hd
Fetching	**Stuka**	2000.	6 w	hd	Calling in	**Arwen**	2003.	3 w	hd
Fetching	**Tódor**	2003.	6 w	hd	Calling in	**Dodi**	2003.	3 w	hd
Fetching	**Füli**	2003.	6 w	hd	Calling in	**Barnus**	2001.	4 w	hw
Fetching	**Tücsök**	2003.	6 w	hd	Calling in	**Rebeka**	2001.	4 w	hw
Fetching	**Maugli**	2003.	6 w	hd	Calling in	**Jimmy-Joe**	2001.	4 w	hw
Fetching	**Oszkár**	2003.	6 w	hd	Calling in	**Minka**	2001.	4 w	hw
Fetching	**Szofi**	2003.	6 w	hd	Calling in	**Bence**	2002.	4 w	hw
Fetching	**Arwen**	2003.	6 w	hd	Calling in	**Bogi**	2002.	4 w	hw
Fetching	**Dodi**	2003.	6 w	hd	Calling in	**Zed**	2002.	4 w	hw
Fetching	**narancspuli**	2004.	6 w	md	Calling in	**Ursula**	2002.	4 w	hw
Fetching	**barnapuli**	2004.	6 w	md	Calling in	**Tóbiás**	2002.	4 w	hw
Fetching	**pumimokus(**	2004.	6 w	md	Calling in	**Maja**	2002.	4 w	hw
Fetching	**pumimocoN**	2004.	6 w	md	Calling in	**Léna**	2002.	4 w	hw
Fetching	**collie2_Rach**	2004.	6 w	md	Calling in	**Borisz**	2002.	4 w	hw
Fetching	**collie2_Rubi**	2004.	6 w	md	Calling in	**Boróka**	2000.	4 w	hd
Fetching	**collie3_Sulta**	2004.	6 w	md	Calling in	**Zokni**	2000.	4 w	hd
Fetching	**collie3_Shad**	2004.	6 w	md	Calling in	**Stuka**	2000.	4 w	hd

Fetching	**csehfarkas1_**	2004.	6 w	md
Fetching	**csehfarkas1_**	2004.	6 w	md
Fetching	**groenisötétk**	2004.	6 w	md
Fetching	**groenivilkék**	2004.	6 w	md
Fetching	**Barnus**	2001.	9 w	hw
Fetching	**Rebeka**	2001.	9 w	hw
Fetching	**Jimmy-Joe**	2001.	9 w	hw
Fetching	**Minka**	2001.	9 w	hw
Fetching	**Bence**	2002.	9 w	hw
Fetching	**Bogi**	2002.	9 w	hw
Fetching	**Zed**	2002.	9 w	hw
Fetching	**Ursula**	2002.	9 w	hw
Fetching	**Tóbiás**	2002.	9 w	hw
Fetching	**Maja**	2002.	9 w	hw
Fetching	**Zazi**	2002.	9 w	hw
Fetching	**Léna**	2002.	9 w	hw
Fetching	**Borisz**	2002.	9 w	hw
Fetching	**Bodza**	2004.	9 w	hw
Fetching	**Dakota**	2004.	9 w	hw
Fetching	**Wolkó**	2004.	9 w	hw
Fetching	**Boróka**	2000.	9 w	hd
Fetching	**Zokni**	2000.	9 w	hd
Fetching	**Stuka**	2000.	9 w	hd
Fetching	**Tódor**	2003.	9 w	hd
Fetching	**Füli**	2003.	9 w	hd
Fetching	**Tücsök**	2003.	9 w	hd
Fetching	**Maugli**	2003.	9 w	hd
Fetching	**Oszkár**	2003.	9 w	hd
Fetching	**Szofi**	2003.	9 w	hd
Fetching	**Arwen**	2003.	9 w	hd
Fetching	**Dodi**	2003.	9 w	hd
Fetching	**narancspuli**	2004.	9 w	md
Fetching	**barnapuli**	2004.	9 w	md
Fetching	**pumimokus**	2004.	9 w	md
Fetching	**pumimocoN**	2004.	9 w	md
Fetching	**collie2_Rach**	2004.	9 w	md
Fetching	**collie2_Rubi**	2004.	9 w	md
Fetching	**collie3_Sulta**	2004.	9 w	md
Fetching	**collie3_Shad**	2004.	9 w	md
Fetching	**csehfarkas1_**	2004.	9 w	md
Fetching	**csehfarkas1_**	2004.	9 w	md
Fetching	**groenisötétk**	2004.	9 w	md
Fetching	**groenivilkék**	2004.	9 w	md

**Experiment**	**Name**	**Tested**	**Age**	**Group**
Sit	**Bence**	2002.	7 w	hw
Sit	**Bogi**	2002.	7 w	hw
Sit	**Zed**	2002.	7 w	hw
Sit	**Ursula**	2002.	7 w	hw
Sit	**Maja**	2002.	7 w	hw
Sit	**Zazi**	2002.	7 w	hw

Calling in	**Tódor**	2003.	4 w	hd
Calling in	**Füli**	2003.	4 w	hd
Calling in	**Tücsök**	2003.	4 w	hd
Calling in	**Maugli**	2003.	4 w	hd
Calling in	**Oszkár**	2003.	4 w	hd
Calling in	**Szofi**	2003.	4 w	hd
Calling in	**Arwen**	2003.	4 w	hd
Calling in	**Dodi**	2003.	4 w	hd
Calling in	**Barnus**	2001.	5 w	hw
Calling in	**Rebeka**	2001.	5 w	hw
Calling in	**Jimmy-Joe**	2001.	5 w	hw
Calling in	**Minka**	2001.	5 w	hw
Calling in	**Bence**	2002.	5 w	hw
Calling in	**Bogi**	2002.	5 w	hw
Calling in	**Zed**	2002.	5 w	hw
Calling in	**Ursula**	2002.	5 w	hw
Calling in	**Tóbiás**	2002.	5 w	hw
Calling in	**Maja**	2002.	5 w	hw
Calling in	**Zazi**	2002.	5 w	hw
Calling in	**Léna**	2002.	5 w	hw
Calling in	**Boróka**	2000.	5 w	hd
Calling in	**Zokni**	2000.	5 w	hd
Calling in	**Stuka**	2000.	5 w	hd
Calling in	**Tódor**	2003.	5 w	hd
Calling in	**Füli**	2003.	5 w	hd
Calling in	**Tücsök**	2003.	5 w	hd
Calling in	**Maugli**	2003.	5 w	hd
Calling in	**Oszkár**	2003.	5 w	hd
Calling in	**Szofi**	2003.	5 w	hd
Calling in	**Arwen**	2003.	5 w	hd
Calling in	**Dodi**	2003.	5 w	hd
Calling in	**Barnus**	2001.	6 w	hw
Calling in	**Rebeka**	2001.	6 w	hw
Calling in	**Jimmy-Joe**	2001.	6 w	hw
Calling in	**Minka**	2001.	6 w	hw
Calling in	**Bence**	2002.	6 w	hw
Calling in	**Bogi**	2002.	6 w	hw
Calling in	**Zed**	2002.	6 w	hw
Calling in	**Ursula**	2002.	6 w	hw
Calling in	**Tóbiás**	2002.	6 w	hw
Calling in	**Maja**	2002.	6 w	hw
Calling in	**Zazi**	2002.	6 w	hw
Calling in	**Léna**	2002.	6 w	hw
Calling in	**Borisz**	2002.	6 w	hw
Calling in	**Boróka**	2000.	6 w	hd
Calling in	**Stuka**	2000.	6 w	hd
Calling in	**Tódor**	2003.	6 w	hd
Calling in	**Füli**	2003.	6 w	hd
Calling in	**Tücsök**	2003.	6 w	hd
Calling in	**Maugli**	2003.	6 w	hd

Sit	**Léna**	2002.	7 w	hw
Sit	**Borisz**	2002.	7 w	hw
Sit	**Tódor**	2003.	7 w	hd
Sit	**Füli**	2003.	7 w	hd
Sit	**Tücsök**	2003.	7 w	hd
Sit	**Maugli**	2003.	7 w	hd
Sit	**Oszkár**	2003.	7 w	hd
Sit	**Szofi**	2003.	7 w	hd
Sit	**Arwen**	2003.	7 w	hd
Sit	**Dodi**	2003.	7 w	hd
Sit	**Bence**	2002.	9 w	hw
Sit	**Bogi**	2002.	9 w	hw
Sit	**Zed**	2002.	9 w	hw
Sit	**Ursula**	2002.	9 w	hw
Sit	**Maja**	2002.	9 w	hw
Sit	**Zazi**	2002.	9 w	hw
Sit	**Léna**	2002.	9 w	hw
Sit	**Tódor**	2003.	9 w	hd
Sit	**Füli**	2003.	9 w	hd
Sit	**Tücsök**	2003.	9 w	hd
Sit	**Maugli**	2003.	9 w	hd
Sit	**Oszkár**	2003.	9 w	hd
Sit	**Szofi**	2003.	9 w	hd
Sit	**Dodi**	2003.	9 w	hd
Sit	**Barnus**	2001.	12 w	hw
Sit	**Jimmy-Joe**	2001.	12 w	hw
Sit	**Bence**	2002.	12 w	hw
Sit	**Bogi**	2002.	12 w	hw
Sit	**Zed**	2002.	12 w	hw
Sit	**Ursula**	2002.	12 w	hw
Sit	**Tóbiás**	2002.	12 w	hw
Sit	**Maja**	2002.	12 w	hw
Sit	**Zazi**	2002.	12 w	hw
Sit	**Léna**	2002.	12 w	hw
Sit	**Borisz**	2002.	12 w	hw
Sit	**Boróka**	2000.	12 w	hd
Sit	**Zokni**	2000.	12 w	hd
Sit	**Stuka**	2000.	12 w	hd
Sit	**Füli**	2003.	12 w	hd
Sit	**Tücsök**	2003.	12 w	hd
Sit	**Maugli**	2003.	12 w	hd
Sit	**Oszkár**	2003.	12 w	hd
Sit	**Szofi**	2003.	12 w	hd
Sit	**Arwen**	2003.	12 w	hd
Sit	**Dodi**	2003.	12 w	hd
Sit	**Barnus**	2001.	16 w	hw
Sit	**Rebeka**	2001.	16 w	hw
Sit	**Jimmy-Joe**	2001.	16 w	hw
Sit	**Minka**	2001.	16 w	hw
Sit	**Bence**	2002.	16 w	hw

Calling in	**Oszkár**	2003.	6 w	hd
Calling in	**Szofi**	2003.	6 w	hd
Calling in	**Arwen**	2003.	6 w	hd
Calling in	**Dodi**	2003.	6 w	hd
Calling in	**narancspuli**	2004.	6 w	md
Calling in	**barnapuli**	2004.	6 w	md
Calling in	**pumimokusC**	2004.	6 w	md
Calling in	**pumimocoN**	2004.	6 w	md
Calling in	**collie2_Rache**	2004.	6 w	md
Calling in	**collie2_Rubin**	2004.	6 w	md
Calling in	**collie3_Sultan**	2004.	6 w	md
Calling in	**collie3_Shado**	2004.	6 w	md
Calling in	**csehfarkas1_f**	2004.	6 w	md
Calling in	**csehfarkas1_l**	2004.	6 w	md
Calling in	**groenisötétké**	2004.	6 w	md
Calling in	**groenivilkék5**	2004.	6 w	md
Calling in	**Bence**	2002.	7 w	hw
Calling in	**Bogi**	2002.	7 w	hw
Calling in	**Zed**	2002.	7 w	hw
Calling in	**Ursula**	2002.	7 w	hw
Calling in	**Tóbiás**	2002.	7 w	hw
Calling in	**Maja**	2002.	7 w	hw
Calling in	**Zazi**	2002.	7 w	hw
Calling in	**Léna**	2002.	7 w	hw
Calling in	**Borisz**	2002.	7 w	hw
Calling in	**Tódor**	2003.	7 w	hd
Calling in	**Füli**	2003.	7 w	hd
Calling in	**Tücsök**	2003.	7 w	hd
Calling in	**Maugli**	2003.	7 w	hd
Calling in	**Oszkár**	2003.	7 w	hd
Calling in	**Szofi**	2003.	7 w	hd
Calling in	**Arwen**	2003.	7 w	hd
Calling in	**Dodi**	2003.	7 w	hd
Calling in	**Bence**	2002.	8 w	hw
Calling in	**Bogi**	2002.	8 w	hw
Calling in	**Zed**	2002.	8 w	hw
Calling in	**Ursula**	2002.	8 w	hw
Calling in	**Tóbiás**	2002.	8 w	hw
Calling in	**Maja**	2002.	8 w	hw
Calling in	**Zazi**	2002.	8 w	hw
Calling in	**Léna**	2002.	8 w	hw
Calling in	**Borisz**	2002.	8 w	hw
Calling in	**Tódor**	2003.	8 w	hd
Calling in	**Füli**	2003.	8 w	hd
Calling in	**Tücsök**	2003.	8 w	hd
Calling in	**Maugli**	2003.	8 w	hd
Calling in	**Oszkár**	2003.	8 w	hd
Calling in	**Szofi**	2003.	8 w	hd
Calling in	**Arwen**	2003.	8 w	hd
Calling in	**Dodi**	2003.	8 w	hd

Sit	**Bogi**	2002.	16 w	hw
Sit	**Zed**	2002.	16 w	hw
Sit	**Ursula**	2002.	16 w	hw
Sit	**Maja**	2002.	16 w	hw
Sit	**Zazi**	2002.	16 w	hw
Sit	**Léna**	2002.	16 w	hw
Sit	**Borisz**	2002.	16 w	hw
Sit	**Boróka**	2000.	16 w	hd
Sit	**Tódor**	2003.	16 w	hd
Sit	**Füli**	2003.	16 w	hd
Sit	**Tücsök**	2003.	16 w	hd
Sit	**Oszkár**	2003.	16 w	hd
Sit	**Arwen**	2003.	16 w	hd
Sit	**Barnus**	2001.	24 w	hw
Sit	**Rebeka**	2001.	24 w	hw
Sit	**Jimmy-Joe**	2001.	24 w	hw
Sit	**Minka**	2001.	24 w	hw
Sit	**Bence**	2002.	24 w	hw
Sit	**Bogi**	2002.	24 w	hw
Sit	**Ursula**	2002.	24 w	hw
Sit	**Maja**	2002.	24 w	hw
Sit	**Boróka**	2000.	24 w	hd
Sit	**Zokni**	2000.	24 w	hd
Sit	**Stuka**	2000.	24 w	hd
Sit	**Tódor**	2003.	24 w	hd
Sit	**Füli**	2003.	24 w	hd
Sit	**Tücsök**	2003.	24 w	hd
Sit	**Maugli**	2003.	24 w	hd
Sit	**Oszkár**	2003.	24 w	hd
Sit	**Szofi**	2003.	24 w	hd
Sit	**Dodi**	2003.	24 w	hd

**Experiment**	**Name**	**Tested**	**Age**	**Group**
Brushing	**Barnus**	2001.	12 w	hw
Brushing	**Jimmy-Joe**	2001.	12 w	hw
Brushing	**Bence**	2002.	12 w	hw
Brushing	**Bogi**	2002.	12 w	hw
Brushing	**Zed**	2002.	12 w	hw
Brushing	**Ursula**	2002.	12 w	hw
Brushing	**Tóbiás**	2002.	12 w	hw
Brushing	**Maja**	2002.	12 w	hw
Brushing	**Zazi**	2002.	12 w	hw
Brushing	**Léna**	2002.	12 w	hw
Brushing	**Borisz**	2002.	12 w	hw
Brushing	**Boróka**	2000.	12 w	hd
Brushing	**Zokni**	2000.	12 w	hd
Brushing	**Stuka**	2000.	12 w	hd
Brushing	**Füli**	2003.	12 w	hd
Brushing	**Tücsök**	2003.	12 w	hd
Brushing	**Maugli**	2003.	12 w	hd
Brushing	**Oszkár**	2003.	12 w	hd

Calling in	**narancspuli**	2004.	8 w	md
Calling in	**barnapuli**	2004.	8 w	md
Calling in	**pumimokusC**	2004.	8 w	md
Calling in	**pumimocoN**	2004.	8 w	md
Calling in	**collie2_Rache**	2004.	8 w	md
Calling in	**collie2_Rubin**	2004.	8 w	md
Calling in	**collie3_Sultan**	2004.	8 w	md
Calling in	**collie3_Shado**	2004.	8 w	md
Calling in	**csehfarkas1_f**	2004.	8 w	md
Calling in	**csehfarkas1_l**	2004.	8 w	md
Calling in	**groenisötétké**	2004.	8 w	md
Calling in	**groenivilkék5**	2004.	8 w	md
Calling in	**Barnus**	2001.	12 w	hw
Calling in	**Rebeka**	2001.	12 w	hw
Calling in	**Jimmy-Joe**	2001.	12 w	hw
Calling in	**Minka**	2001.	12 w	hw
Calling in	**Bence**	2002.	12 w	hw
Calling in	**Bogi**	2002.	12 w	hw
Calling in	**Zed**	2002.	12 w	hw
Calling in	**Ursula**	2002.	12 w	hw
Calling in	**Tóbiás**	2002.	12 w	hw
Calling in	**Maja**	2002.	12 w	hw
Calling in	**Zazi**	2002.	12 w	hw
Calling in	**Léna**	2002.	12 w	hw
Calling in	**Borisz**	2002.	12 w	hw
Calling in	**Boróka**	2000.	12 w	hd
Calling in	**Zokni**	2000.	12 w	hd
Calling in	**Stuka**	2000.	12 w	hd
Calling in	**Tódor**	2003.	12 w	hd
Calling in	**Füli**	2003.	12 w	hd
Calling in	**Tücsök**	2003.	12 w	hd
Calling in	**Maugli**	2003.	12 w	hd
Calling in	**Oszkár**	2003.	12 w	hd
Calling in	**Szofi**	2003.	12 w	hd
Calling in	**Arwen**	2003.	12 w	hd
Calling in	**Dodi**	2003.	12 w	hd
Calling in	**Barnus**	2001.	16 w	hw
Calling in	**Rebeka**	2001.	16 w	hw
Calling in	**Jimmy-Joe**	2001.	16 w	hw
Calling in	**Minka**	2001.	16 w	hw
Calling in	**Bence**	2002.	16 w	hw
Calling in	**Bogi**	2002.	16 w	hw
Calling in	**Ursula**	2002.	16 w	hw
Calling in	**Tóbiás**	2002.	16 w	hw
Calling in	**Léna**	2002.	16 w	hw
Calling in	**Tódor**	2003.	16 w	hd
Calling in	**Füli**	2003.	16 w	hd
Calling in	**Tücsök**	2003.	16 w	hd
Calling in	**Maugli**	2003.	16 w	hd
Calling in	**Szofi**	2003.	16 w	hd

Brushing	Szofi	2003.	12 w	hd
Brushing	Arwen	2003.	12 w	hd
Brushing	Dodi	2003.	12 w	hd
Brushing	Barnus	2001.	16 w	hw
Brushing	Rebeka	2001.	16 w	hw
Brushing	Jimmy-Joe	2001.	16 w	hw
Brushing	Minka	2001.	16 w	hw
Brushing	Bence	2002.	16 w	hw
Brushing	Bogi	2002.	16 w	hw
Brushing	Zed	2002.	16 w	hw
Brushing	Ursula	2002.	16 w	hw
Brushing	Tóbiás	2002.	16 w	hw
Brushing	Maja	2002.	16 w	hw
Brushing	Zazi	2002.	16 w	hw
Brushing	Léna	2002.	16 w	hw
Brushing	Borisz	2002.	16 w	hw
Brushing	Boróka	2000.	16 w	hd
Brushing	Zokni	2000.	16 w	hd
Brushing	Stuka	2000.	16 w	hd
Brushing	Tódor	2003.	16 w	hd
Brushing	Füli	2003.	16 w	hd
Brushing	Tücsök	2003.	16 w	hd
Brushing	Oszkár	2003.	16 w	hd
Brushing	Arwen	2003.	16 w	hd

Experiment	Name	Tested	Age	Group
Muzzle	Barnus	2001.	16 w	hw
Muzzle	Rebeka	2001.	16 w	hw
Muzzle	Jimmy-Joe	2001.	16 w	hw
Muzzle	Minka	2001.	16 w	hw
Muzzle	Bence	2002.	16 w	hw
Muzzle	Bogi	2002.	16 w	hw
Muzzle	Zed	2002.	16 w	hw
Muzzle	Ursula	2002.	16 w	hw
Muzzle	Tóbiás	2002.	16 w	hw
Muzzle	Maja	2002.	16 w	hw
Muzzle	Zazi	2002.	16 w	hw
Muzzle	Léna	2002.	16 w	hw
Muzzle	Borisz	2002.	16 w	hw
Muzzle	Boróka	2000.	16 w	hd
Muzzle	Zokni	2000.	16 w	hd
Muzzle	Stuka	2000.	16 w	hd
Muzzle	Tódor	2003.	16 w	hd
Muzzle	Füli	2003.	16 w	hd
Muzzle	Tücsök	2003.	16 w	hd
Muzzle	Oszkár	2003.	16 w	hd
Muzzle	Arwen	2003.	16 w	hd
Muzzle	Barnus	2001.	24 w	hw
Muzzle	Rebeka	2001.	24 w	hw
Muzzle	Jimmy-Joe	2001.	24 w	hw
Muzzle	Minka	2001.	24 w	hw

Calling in	Dodi	2003.	16 w	hd
Calling in	Barnus	2001.	24 w	hw
Calling in	Rebeka	2001.	24 w	hw
Calling in	Jimmy-Joe	2001.	24 w	hw
Calling in	Minka	2001.	24 w	hw
Calling in	Bence	2002.	24 w	hw
Calling in	Bogi	2002.	24 w	hw
Calling in	Ursula	2002.	24 w	hw
Calling in	Maja	2002.	24 w	hw
Calling in	Boróka	2000.	24 w	hd
Calling in	Zokni	2000.	24 w	hd
Calling in	Stuka	2000.	24 w	hd
Calling in	Tódor	2003.	24 w	hd
Calling in	Füli	2003.	24 w	hd
Calling in	Tücsök	2003.	24 w	hd
Calling in	Maugli	2003.	24 w	hd
Calling in	Oszkár	2003.	24 w	hd
Calling in	Szofi	2003.	24 w	hd
Calling in	Dodi	2003.	24 w	hd

Muzzle	**Bence**	**2002.**	24 w	hw
Muzzle	**Bogi**	**2002.**	24 w	hw
Muzzle	**Ursula**	**2002.**	24 w	hw
Muzzle	**Maja**	**2002.**	24 w	hw
Muzzle	**Boróka**	**2000.**	24 w	hd
Muzzle	**Zokni**	**2000.**	24 w	hd
Muzzle	**Stuka**	**2000.**	24 w	hd
Muzzle	**Tódor**	**2003.**	24 w	hd
Muzzle	**Füli**	**2003.**	24 w	hd
Muzzle	**Tücsök**	**2003.**	24 w	hd
Muzzle	**Oszkár**	**2003.**	24 w	hd
Muzzle	**Szofi**	**2003.**	24 w	hd
Muzzle	**Dodi**	**2003.**	24 w	hd
Muzzle	**Szuzi**	**2003.**	24 w	hd
