# Supplementary material for: Comparing the tractability of young hand-raised wolves (Canis lupus) and dogs (Canis familiaris)
Source: Sci Rep. 2020 Sep 7;10:14678. doi: 10.1038/s41598-020-71687-3 (PMC7477132; doi:10.1038/s41598-020-71687-3)
Supplement: Supplementary file 2 — Supplementary Information 2. [file 41598_2020_71687_MOESM2_ESM.pdf]

**Comparing the tractability of young hand-raised wolves (*Canis lupus*) and dogs (Canis familiaris)**

Dorottya Júlia Ujfalussy [2*], Zsófia Virányi [3, 4], Márta Gácsi [1, 2], Tamás Faragó [2],
Ákos Pogány 2, Boróka Mária Bereczky 1, Ádám Miklósi 2, Enikő Kubinyi 2

*1 MTA-ELTE Comparative Ethology Research Group*
*2 Department of Ethology, ELTE Eötvös Loránd University, Budapest*
*3 Comparative Cognition, Messerli Research Institute,*
*University of Veterinary Medicine, Vienna, Medical University of Vienna, University of Vienna*
*4 Wolf Science Center, Domestication Lab, Konrad Lorenz Institute of Ethology,*
*University of Veterinary Medicine Vienna, Vienna, Austria*

## Supplementary material 2 - All data

**Brushing**

Group 1 -hw
Group 2 -hd

| AgeWeek | Group | Name | Moving Score (0-3) | Biting (number/30 sec) |
|---|---|---|---|---|
| 12 | 1 | **Barnus** | 2 | 5 |
| 12 | 1 | **Jimmy-Joe** | 2 | 2 |
| 12 | 1 | **Bence** | 1 | 1 |
| 12 | 1 | **Bogi** | 1 | 1 |
| 12 | 1 | **Zed** | 1 | 7 |
| 12 | 1 | **Ursula** | 1 | 6 |
| 12 | 1 | **Tóbiás** | 1 | 0 |
| 12 | 1 | **Maja** | 2 | 3 |
| 12 | 1 | **Zazi** | 1 | 3 |
| 12 | 1 | **Léna** | 1 | 0 |
| 12 | 1 | **Borisz** | 1 | 7 |
| 12 | 2 | **Boróka** | 1 | 0 |
| 12 | 2 | **Zokni** | 0 | 0 |
| 12 | 2 | **Stuka** | 1 | 0 |
| 12 | 2 | **Füli** | 0 | 0 |
| 12 | 2 | **Tücsök** | 3 | 1 |
| 12 | 2 | **Maugli** | 2 | 0 |
| 12 | 2 | **Oszkár** | 0 | 0 |
| 12 | 2 | **Szofi** | 3 | 0 |
| 12 | 2 | **Arwen** | 0 | 0 |
| 12 | 2 | **Dodi** | 1 | 2 |
| 16 | 1 | **Barnus** | 2 | 3 |
| 16 | 1 | **Rebeka** | 2 | 3 |
| 16 | 1 | **Jimmy-Joe** | 2 | 2 |
| 16 | 1 | **Minka** | 1 | 5 |
| 16 | 1 | **Bence** | 0 | 4 |
| 16 | 1 | **Bogi** | 1 | 1 |
| 16 | 1 | **Zed** | 1 | 1 |
| 16 | 1 | **Ursula** | 2 | 19 |
| 16 | 1 | **Tóbiás** | 1 | 2 |
| 16 | 1 | **Maja** | 2 | 9 |
| 16 | 1 | **Zazi** | 1 | 3 |

| | | | | |
|---|---|---|---|---|
| 16 | 1 | **Léna** | 1 | 2 |
| 16 | 1 | **Borisz** | 1 | 1 |
| 16 | 2 | **Boróka** | 1 | 0 |
| 16 | 2 | **Zokni** | 3 | 10 |
| 16 | 2 | **Stuka** | 1 | 1 |
| 16 | 2 | **Tódor** | 3 | 2 |
| 16 | 2 | **Füli** | 2 | 0 |
| 16 | 2 | **Tücsök** | 3 | 6 |
| 16 | 2 | **Oszkár** | 1 | 11 |
| 16 | 2 | **Arwen** | 2 | 0 |

**Calling**

Group 1 -hw

Group 2 -hd

Group 3 -md

| AgeWeek | Group | Name | AvLatency | MaxLat |
|---|---|---|---|---|
| 3 | 1 | **Barnus** | 200 | 1 |
| 3 | 1 | **Rebeka** | 12,7 | 0 |
| 3 | 1 | **Minka** | 27,5 | 0 |
| 3 | 1 | **Bence** | 29,6 | 0 |
| 3 | 1 | **Bogi** | 200 | 1 |
| 3 | 1 | **Zed** | 13,6 | 0 |
| 3 | 1 | **Ursula** | 23,9 | 0 |
| 3 | 1 | **Tóbiás** | 37,4 | 0 |
| 3 | 1 | **Maja** | 51,9 | 0 |
| 3 | 1 | **Zazi** | 7,6 | 0 |
| 3 | 1 | **Léna** | 9,2 | 0 |
| 3 | 1 | **Borisz** | 10 | 0 |
| 3 | 2 | **Tódor** | 107,1 | 0 |
| 3 | 2 | **Füli** | 120,8 | 0 |
| 3 | 2 | **Tücsök** | 26,3 | 0 |
| 3 | 2 | **Maugli** | 49,8 | 0 |
| 3 | 2 | **Oszkár** | 24,7 | 0 |
| 3 | 2 | **Szofi** | 106,5 | 0 |
| 3 | 2 | **Arwen** | 200 | 1 |
| 3 | 2 | **Dodi** | 200 | 1 |
| 4 | 1 | **Barnus** | 14,6 | 0 |
| 4 | 1 | **Rebeka** | 5,8 | 0 |
| 4 | 1 | **Jimmy-Joe** | 9,8 | 0 |
| 4 | 1 | **Minka** | 10,8 | 0 |
| 4 | 1 | **Bence** | 108,2 | 0 |
| 4 | 1 | **Bogi** | 200 | 0 |
| 4 | 1 | **Zed** | 63,6 | 0 |
| 4 | 1 | **Ursula** | 14,1 | 0 |
| 4 | 1 | **Tóbiás** | 112,1 | 0 |
| 4 | 1 | **Maja** | 21,6 | 0 |
| 4 | 1 | **Léna** | 142,5 | 0 |
| 4 | 1 | **Borisz** | 32,8 | 0 |
| 4 | 2 | **Boróka** | 1,8 | 0 |

| | | | | |
|---|---|---|---|---|
| 4 | 2 | **Zokni** | 2 | 0 |
| 4 | 2 | **Stuka** | 7,1 | 0 |
| 4 | 2 | **Tódor** | 2,8 | 0 |
| 4 | 2 | **Füli** | 107,6 | 0 |
| 4 | 2 | **Tücsök** | 200 | 1 |
| 4 | 2 | **Maugli** | 6,1 | 0 |
| 4 | 2 | **Oszkár** | 107,6 | 0 |
| 4 | 2 | **Szofi** | 107,3 | 0 |
| 4 | 2 | **Arwen** | 200 | 1 |
| 4 | 2 | **Dodi** | 200 | 1 |
| 5 | 1 | **Barnus** | 200 | 1 |
| 5 | 1 | **Rebeka** | 18,6 | 0 |
| 5 | 1 | **Jimmy-Joe** | 44,2 | 0 |
| 5 | 1 | **Minka** | 10,8 | 0 |
| 5 | 1 | **Bence** | 4,9 | 0 |
| 5 | 1 | **Bogi** | 5 | 0 |
| 5 | 1 | **Zed** | 18,1 | 0 |
| 5 | 1 | **Ursula** | 5,2 | 0 |
| 5 | 1 | **Tóbiás** | 31,1 | 0 |
| 5 | 1 | **Maja** | 5,2 | 0 |
| 5 | 1 | **Zazi** | 12,6 | 0 |
| 5 | 1 | **Léna** | 35,9 | 0 |
| 5 | 2 | **Boróka** | 6,4 | 0 |
| 5 | 2 | **Zokni** | 28,2 | 0 |
| 5 | 2 | **Stuka** | 3,6 | 0 |
| 5 | 2 | **Tódor** | 4,6 | 0 |
| 5 | 2 | **Füli** | 6,8 | 0 |
| 5 | 2 | **Tücsök** | 37,8 | 0 |
| 5 | 2 | **Maugli** | 2,1 | 0 |
| 5 | 2 | **Oszkár** | 104,9 | 0 |
| 5 | 2 | **Szofi** | 5,8 | 0 |
| 5 | 2 | **Arwen** | 12,7 | 0 |
| 5 | 2 | **Dodi** | 3,3 | 0 |
| 6 | 1 | **Barnus** | 40 | 1 |
| 6 | 1 | **Rebeka** | 7 | 0 |
| 6 | 1 | **Jimmy-Joe** | 40 | 1 |
| 6 | 1 | **Minka** | 4 | 0 |
| 6 | 1 | **Bence** | 4 | 0 |
| 6 | 1 | **Bogi** | 40 | 1 |
| 6 | 1 | **Zed** | 4 | 0 |
| 6 | 1 | **Ursula** | 3 | 0 |
| 6 | 1 | **Tóbiás** | 7 | 0 |
| 6 | 1 | **Maja** | 3 | 0 |
| 6 | 1 | **Zazi** | 23 | 0 |
| 6 | 1 | **Léna** | 6 | 0 |
| 6 | 1 | **Borisz** | 24 | 0 |
| 6 | 2 | **Boróka** | 6 | 0 |
| 6 | 2 | **Stuka** | 6 | 0 |
| 6 | 2 | **Tódor** | 5 | 0 |
| 6 | 2 | **Füli** | 7 | 0 |

| | | | | |
|---|---|---|---|---|
| 6 | 2 | Tücsök | 3 | 0 |
| 6 | 2 | Maugli | 3 | 0 |
| 6 | 2 | Oszkár | 40 | 1 |
| 6 | 2 | Szofi | 40 | 1 |
| 6 | 2 | Arwen | 40 | 1 |
| 6 | 2 | Dodi | 5 | 0 |
| 6 | 3 | narancspuli | 25 | 0 |
| 6 | 3 | barnapuli | 4 | 0 |
| 6 | 3 | pumimokusC | 13 | 0 |
| 6 | 3 | pumimocoN | 9 | 0 |
| 6 | 3 | collie2_Rachel | 40 | 1 |
| 6 | 3 | collie2_Rubin | 40 | 1 |
| 6 | 3 | collie3_Sultan | 7 | 0 |
| 6 | 3 | collie3_Shadow | 11 | 0 |
| 6 | 3 | csehfarkas1_fel | 40 | 1 |
| 6 | 3 | csehfarkas1_lila | 9 | 0 |
| 6 | 3 | groenisötétkék | 40 | 1 |
| 6 | 3 | groenivilkék5 | 40 | 1 |
| 7 | 1 | Bence | 6,9 | 0 |
| 7 | 1 | Bogi | 5,9 | 0 |
| 7 | 1 | Zed | 3,4 | 0 |
| 7 | 1 | Ursula | 5,7 | 0 |
| 7 | 1 | Tóbiás | 35,4 | 0 |
| 7 | 1 | Maja | 3,3 | 0 |
| 7 | 1 | Zazi | 115,3 | 0 |
| 7 | 1 | Léna | 109,4 | 0 |
| 7 | 1 | Borisz | 23,4 | 0 |
| 7 | 2 | Tódor | 5,3 | 0 |
| 7 | 2 | Füli | 3,73 | 0 |
| 7 | 2 | Tücsök | 4,7 | 0 |
| 7 | 2 | Maugli | 8,4 | 0 |
| 7 | 2 | Oszkár | 4,3 | 0 |
| 7 | 2 | Szofi | 8,9 | 0 |
| 7 | 2 | Arwen | 12,5 | 0 |
| 7 | 2 | Dodi | 42,4 | 0 |
| 8 | 1 | Bence | 3 | 0 |
| 8 | 1 | Bogi | 3 | 0 |
| 8 | 1 | Zed | 3 | 0 |
| 8 | 1 | Ursula | 3 | 0 |
| 8 | 1 | Tóbiás | 5 | 0 |
| 8 | 1 | Maja | 17 | 0 |
| 8 | 1 | Zazi | 5 | 0 |
| 8 | 1 | Léna | 3 | 0 |
| 8 | 1 | Borisz | 40 | 1 |
| 8 | 2 | Tódor | 11 | 0 |
| 8 | 2 | Füli | 5 | 0 |
| 8 | 2 | Tücsök | 32 | 0 |
| 8 | 2 | Maugli | 3 | 0 |
| 8 | 2 | Oszkár | 4 | 0 |
| 8 | 2 | Szofi | 5 | 0 |

| | | | | |
|---|---|---|---|---|
| 8 | 2 | **Arwen** | 40 | 1 |
| 8 | 2 | **Dodi** | 4 | 0 |
| 8 | 3 | **narancspuli** | 40 | 1 |
| 8 | 3 | **barnapuli** | 40 | 1 |
| 8 | 3 | **pumimokusC** | 7 | 0 |
| 8 | 3 | **pumimocoN** | 3 | 0 |
| 8 | 3 | **collie2_Rachel** | 2 | 0 |
| 8 | 3 | **collie2_Rubin** | 9 | 0 |
| 8 | 3 | **collie3_Sultan** | 3 | 0 |
| 8 | 3 | **collie3_Shadow** | 3 | 0 |
| 8 | 3 | **csehfarkas1_feh** | 3 | 0 |
| 8 | 3 | **csehfarkas1_lila** | 13 | 0 |
| 8 | 3 | **groenisötétkék** | 5 | 0 |
| 8 | 3 | **groenivilkék5** | 2 | 0 |
| 12 | 1 | **Barnus** | 9,4 | 0 |
| 12 | 1 | **Rebeka** | 22,2 | 0 |
| 12 | 1 | **Jimmy-Joe** | 8,05 | 0 |
| 12 | 1 | **Minka** | 74,75 | 0 |
| 12 | 1 | **Bence** | 13 | 0 |
| 12 | 1 | **Bogi** | 200 | 0 |
| 12 | 1 | **Zed** | 28,8 | 0 |
| 12 | 1 | **Ursula** | 125,4 | 0 |
| 12 | 1 | **Tóbiás** | 28,5 | 0 |
| 12 | 1 | **Maja** | 10,3 | 0 |
| 12 | 1 | **Zazi** | 14,2 | 0 |
| 12 | 1 | **Léna** | 8,1 | 0 |
| 12 | 1 | **Borisz** | 104,6 | 0 |
| 12 | 2 | **Boróka** | 101,00 | 0 |
| 12 | 2 | **Zokni** | 2,90 | 0 |
| 12 | 2 | **Stuka** | 12,50 | 0 |
| 12 | 2 | **Tódor** | 9,20 | 0 |
| 12 | 2 | **Füli** | 5,30 | 0 |
| 12 | 2 | **Tücsök** | 7,10 | 0 |
| 12 | 2 | **Maugli** | 8,40 | 0 |
| 12 | 2 | **Oszkár** | 10,00 | 0 |
| 12 | 2 | **Szofi** | 43,40 | 0 |
| 12 | 2 | **Arwen** | 14,07 | 0 |
| 12 | 2 | **Dodi** | 48,90 | 0 |
| 16 | 1 | **Barnus** | 33,3 | 0 |
| 16 | 1 | **Rebeka** | 40,3 | 0 |
| 16 | 1 | **Jimmy-Joe** | 17,3 | 0 |
| 16 | 1 | **Minka** | 21,8 | 0 |
| 16 | 1 | **Bence** | 9,3 | 0 |
| 16 | 1 | **Bogi** | 119,2 | 0 |
| 16 | 1 | **Ursula** | 11,4 | 0 |
| 16 | 1 | **Tóbiás** | 4,9 | 0 |
| 16 | 1 | **Léna** | 7,8 | 0 |
| 16 | 2 | **Tódor** | 7,07 | 0 |
| 16 | 2 | **Füli** | 6,80 | 0 |
| 16 | 2 | **Tücsök** | 5,70 | 0 |

| | | | | |
|---|---|---|---|---|
| 16 | 2 | **Maugli** | 3,10 | 0 |
| 16 | 2 | **Szofi** | 8,40 | 0 |
| 16 | 2 | **Dodi** | 19,60 | 0 |
| 24 | 1 | **Barnus** | 200,00 | 1 |
| 24 | 1 | **Rebeka** | 62,80 | 0 |
| 24 | 1 | **Jimmy-Joe** | 200,00 | 1 |
| 24 | 1 | **Minka** | 200,00 | 1 |
| 24 | 1 | **Bence** | 7,00 | 0 |
| 24 | 1 | **Bogi** | 9,10 | 0 |
| 24 | 1 | **Ursula** | 11,60 | 0 |
| 24 | 1 | **Maja** | 140,20 | 0 |
| 24 | 2 | **Boróka** | 42,80 | 0 |
| 24 | 2 | **Zokni** | 12,40 | 0 |
| 24 | 2 | **Stuka** | 8,40 | 0 |
| 24 | 2 | **Tódor** | 6,85 | 0 |
| 24 | 2 | **Füli** | 11,50 | 0 |
| 24 | 2 | **Tücsök** | 4,80 | 0 |
| 24 | 2 | **Maugli** | 38,60 | 0 |
| 24 | 2 | **Oszkár** | 7,87 | 0 |
| 24 | 2 | **Szofi** | 51,90 | 0 |
| 24 | 2 | **Dodi** | 48,60 | 0 |

**Muzzle**

Group 1 -hw

Group 2 -hd

| **AgeWeek** | **Group** | **Name** | **put on muzzle (0-3)** | **moving (0-3)** |
|---|---|---|---|---|
| 16 | 1 | **Barnus** | 2 | 2 |
| 16 | 1 | **Rebeka** | 1 | 1 |
| 16 | 1 | **Jimmy-Joe** | 3 | 2 |
| 16 | 1 | **Minka** | 0 | 1 |
| 16 | 1 | **Bence** | 0 | 1 |
| 16 | 1 | **Bogi** | 1 | 1 |
| 16 | 1 | **Zed** | 0 | 2 |
| 16 | 1 | **Ursula** | 1 | 2 |
| 16 | 1 | **Tóbiás** | 0 | 2 |
| 16 | 1 | **Maja** | 2 | 1 |
| 16 | 1 | **Zazi** | 0 | 2 |
| 16 | 1 | **Léna** | 0 | 2 |
| 16 | 1 | **Borisz** | 0 | 2 |
| 16 | 2 | **Boróka** | 0 | 2 |
| 16 | 2 | **Zokni** | 0 | 2 |
| 16 | 2 | **Stuka** | 0 | 3 |
| 16 | 2 | **Tódor** | 0 | 1 |
| 16 | 2 | **Füli** | 1 | 1 |
| 16 | 2 | **Tücsök** | 3 | 0 |
| 16 | 2 | **Oszkár** | 0 | 2 |
| 16 | 2 | **Arwen** | 0 | 1 |
| 24 | 1 | **Barnus** | 3 | 3 |
| 24 | 1 | **Rebeka** | 0 | 2 |
| 24 | 1 | **Jimmy-Joe** | 3 | 0 |

| | | | | |
|---|---|---|---|---|
| 24 | 1 | **Minka** | 1 | 2 |
| 24 | 1 | **Bence** | 0 | 1 |
| 24 | 1 | **Bogi** | 0 | 1 |
| 24 | 1 | **Ursula** | 0 | 3 |
| 24 | 1 | **Maja** | 0 | 2 |
| 24 | 2 | **Boróka** | 3 | 2 |
| 24 | 2 | **Zokni** | 0 | 3 |
| 24 | 2 | **Stuka** | 1 | 3 |
| 24 | 2 | **Tódor** | 0 | 3 |
| 24 | 2 | **Füli** | 0 | 2 |
| 24 | 2 | **Tücsök** | 3 | 2 |
| 24 | 2 | **Oszkár** | 0 | 3 |
| 24 | 2 | **Szofi** | 1 | 3 |
| 24 | 2 | **Dodi** | 1 | 3 |
| 24 | 2 | **Szuzi** | 0 | 3 |

**Sitting**

Group 1 -hw

Group 2 -hd

| AgeWeek | Group | Name | Latency | SV |
|---|---|---|---|---|
| 7 | 1 | **Bence** | 69,0 | 1 |
| 7 | 1 | **Bogi** | 52,2 | 1 |
| 7 | 1 | **Zed** | 285,7 | 1 |
| 7 | 1 | **Ursula** | 45,6 | 1 |
| 7 | 1 | **Maja** | 296,3 | 1 |
| 7 | 1 | **Zazi** | 74,2 | 1 |
| 7 | 1 | **Léna** | 74,0 | 1 |
| 7 | 1 | **Borisz** | 289,7 | 1 |
| 7 | 2 | **Tódor** | 12,8 | 1 |
| 7 | 2 | **Füli** | 45,0 | 1 |
| 7 | 2 | **Tücsök** | 28,0 | 1 |
| 7 | 2 | **Maugli** | 64,2 | 1 |
| 7 | 2 | **Oszkár** | 90,5 | 1 |
| 7 | 2 | **Szofi** | 96,2 | 1 |
| 7 | 2 | **Arwen** | 21,0 | 1 |
| 7 | 2 | **Dodi** | 67,2 | 1 |
| 9 | 1 | **Bence** | 110,8 | 1 |
| 9 | 1 | **Bogi** | 79,0 | 1 |
| 9 | 1 | **Zed** | 19,0 | 1 |
| 9 | 1 | **Ursula** | 43,4 | 1 |
| 9 | 1 | **Maja** | 33,5 | 1 |
| 9 | 1 | **Zazi** | 252,6 | 1 |
| 9 | 1 | **Léna** | 94,0 | 1 |
| 9 | 2 | **Tódor** | 61,6 | 1 |
| 9 | 2 | **Füli** | 155,8 | 1 |
| 9 | 2 | **Tücsök** | 112,7 | 1 |
| 9 | 2 | **Maugli** | 132,4 | 1 |
| 9 | 2 | **Oszkár** | 127,1 | 1 |
| 9 | 2 | **Szofi** | 61,9 | 1 |
| 9 | 2 | **Dodi** | 182,6 | 1 |

| | | | | |
|---|---|---|---|---|
| 12 | 1 | **Barnus** | 233,3 | 1 |
| 12 | 1 | **Jimmy-Joe** | 78,4 | 1 |
| 12 | 1 | **Bence** | 64,5 | 1 |
| 12 | 1 | **Bogi** | 7,4 | 1 |
| 12 | 1 | **Zed** | 24,0 | 1 |
| 12 | 1 | **Ursula** | 73,4 | 1 |
| 12 | 1 | **Tóbiás** | 81,1 | 1 |
| 12 | 1 | **Maja** | 94,6 | 1 |
| 12 | 1 | **Zazi** | 78,2 | 1 |
| 12 | 1 | **Léna** | 170,0 | 1 |
| 12 | 1 | **Borisz** | 116,1 | 1 |
| 12 | 2 | **Boróka** | 69,2 | 1 |
| 12 | 2 | **Zokni** | 142,0 | 1 |
| 12 | 2 | **Stuka** | 66,8 | 1 |
| 12 | 2 | **Füli** | 186,1 | 1 |
| 12 | 2 | **Tücsök** | 214,6 | 1 |
| 12 | 2 | **Maugli** | 73,0 | 1 |
| 12 | 2 | **Oszkár** | 93,9 | 1 |
| 12 | 2 | **Szofi** | 62,4 | 1 |
| 12 | 2 | **Arwen** | 212,9 | 1 |
| 12 | 2 | **Dodi** | 67,6 | 1 |
| 16 | 1 | **Barnus** | 214,9 | 1 |
| 16 | 1 | **Rebeka** | 17,8 | 1 |
| 16 | 1 | **Jimmy-Joe** | 128,0 | 1 |
| 16 | 1 | **Minka** | 23,8 | 1 |
| 16 | 1 | **Bence** | 107,6 | 1 |
| 16 | 1 | **Bogi** | 65,0 | 1 |
| 16 | 1 | **Zed** | 161,2 | 1 |
| 16 | 1 | **Ursula** | 87,4 | 1 |
| 16 | 1 | **Maja** | 112,5 | 1 |
| 16 | 1 | **Zazi** | 93,6 | 1 |
| 16 | 1 | **Léna** | 278,8 | 1 |
| 16 | 1 | **Borisz** | 78,4 | 1 |
| 16 | 2 | **Boróka** | 29,6 | 1 |
| 16 | 2 | **Tódor** | 58,8 | 1 |
| 16 | 2 | **Füli** | 44,8 | 1 |
| 16 | 2 | **Tücsök** | 35,0 | 1 |
| 16 | 2 | **Oszkár** | 29,4 | 1 |
| 16 | 2 | **Arwen** | 151,4 | 1 |
| 24 | 1 | **Barnus** | 72,8 | 1 |
| 24 | 1 | **Rebeka** | 51,0 | 1 |
| 24 | 1 | **Jimmy-Joe** | 122,4 | 1 |
| 24 | 1 | **Minka** | 56,0 | 1 |
| 24 | 1 | **Bence** | 73,5 | 1 |
| 24 | 1 | **Bogi** | 44,8 | 1 |
| 24 | 1 | **Ursula** | 171,4 | 1 |
| 24 | 1 | **Maja** | 74,1 | 1 |
| 24 | 2 | **Boróka** | 68,0 | 1 |
| 24 | 2 | **Zokni** | 93,0 | 1 |
| 24 | 2 | **Stuka** | 68,0 | 1 |

| | | | | |
|---|---|---|---|---|
| 24 | 2 | **Tódor** | 4,8 | 1 |
| 24 | 2 | **Füli** | 76,4 | 1 |
| 24 | 2 | **Tücsök** | 87,2 | 1 |
| 24 | 2 | **Maugli** | 64,0 | 1 |
| 24 | 2 | **Oszkár** | 87,8 | 1 |
| 24 | 2 | **Szofi** | 91,8 | 1 |
| 24 | 2 | **Dodi** | 6,8 | 1 |

**Fetching**

Group 1 -hw

Group 2 -hd

Group 3 -md

| **AgeWeek** | **Group** | **Name** | retrieving | aggressive | carry away |
|---|---|---|---|---|---|
| 6 | 1 | **Barnus** | 0 | 0 | 0 |
| 6 | 1 | **Rebeka** | 0 | 0 | 3 |
| 6 | 1 | **Jimmy-Joe** | 0 | 3 | 0 |
| 6 | 1 | **Minka** | 0 | 1 | 2 |
| 6 | 1 | **Bence** | 0 | 1 | 0 |
| 6 | 1 | **Bogi** | 0 | 0 | 0 |
| 6 | 1 | **Zed** | 0 | 0 | 0 |
| 6 | 1 | **Ursula** | 1 | 0 | 1 |
| 6 | 1 | **Tóbiás** | 0 | 0 | 0 |
| 6 | 1 | **Maja** | 0 | 0 | 0 |
| 6 | 1 | **Zazi** | 0 | 0 | 0 |
| 6 | 1 | **Léna** | 1 | 0 | 0 |
| 6 | 1 | **Borisz** | 0 | 0 | 0 |
| 6 | 1 | **Bodza** | 0 | 3 | 0 |
| 6 | 1 | **Dakota** | 0 | 0 | 0 |
| 6 | 1 | **Wolkó** | 0 | 0 | 0 |
| 6 | 2 | **Boróka** | 1 | 0 | 0 |
| 6 | 2 | **Zokni** | 1 | 0 | 0 |
| 6 | 2 | **Stuka** | 0 | 0 | 0 |
| 6 | 2 | **Tódor** | 2 | 0 | 0 |
| 6 | 2 | **Füli** | 3 | 0 | 0 |
| 6 | 2 | **Tücsök** | 1 | 0 | 1 |
| 6 | 2 | **Maugli** | 1 | 0 | 0 |
| 6 | 2 | **Oszkár** | 3 | 0 | 0 |
| 6 | 2 | **Szofi** | 0 | 0 | 0 |
| 6 | 2 | **Arwen** | 2 | 0 | 0 |
| 6 | 2 | **Dodi** | 1 | 0 | 0 |
| 6 | 3 | **narancspuli** | 0 | 0 | 0 |
| 6 | 3 | **barnapuli** | 0 | 0 | 0 |
| 6 | 3 | **pumimokusC** | 2 | 0 | 0 |
| 6 | 3 | **pumimocoN** | 1 | 0 | 0 |
| 6 | 3 | **collie2_Rachel** | 2 | 0 | 0 |
| 6 | 3 | **collie2_Rubin** | 1 | 0 | 0 |

| | | | | | |
|---|---|---|---|---|---|
| 6 | 3 | collie3_Sultan | 0 | 0 | 0 |
| 6 | 3 | collie3_Shadow | 0 | 0 | 0 |
| 6 | 3 | csehfarkas1_fel | 0 | 0 | 0 |
| 6 | 3 | csehfarkas1_lila | 0 | 0 | 0 |
| 6 | 3 | groenisötétkék | 1 | 0 | 2 |
| 6 | 3 | groenivilkék5 | 2 | 0 | 0 |
| 9 | 1 | Barnus | 0 | 0 | 2 |
| 9 | 1 | Rebeka | 1 | 0 | 1 |
| 9 | 1 | Jimmy-Joe | 0 | 0 | 3 |
| 9 | 1 | Minka | 0 | 1 | 1 |
| 9 | 1 | Bence | 1 | 0 | 1 |
| 9 | 1 | Bogi | 0 | 0 | 0 |
| 9 | 1 | Zed | 0 | 1 | 3 |
| 9 | 1 | Ursula | 0 | 0 | 2 |
| 9 | 1 | Tóbiás | 0 | 0 | 0 |
| 9 | 1 | Maja | 0 | 2 | 1 |
| 9 | 1 | Zazi | 3 | 0 | 0 |
| 9 | 1 | Léna | 0 | 0 | 1 |
| 9 | 1 | Borisz | 2 | 0 | 0 |
| 9 | 1 | Bodza | 0 | 1 | 2 |
| 9 | 1 | Dakota | 0 | 0 | 0 |
| 9 | 1 | Wolkó | 0 | 0 | 0 |
| 9 | 2 | Boróka | 3 | 0 | 0 |
| 9 | 2 | Zokni | 0 | 0 | 0 |
| 9 | 2 | Stuka | 0 | 0 | 2 |
| 9 | 2 | Tódor | 1 | 0 | 0 |
| 9 | 2 | Füli | 2 | 0 | 2 |
| 9 | 2 | Tücsök | 3 | 0 | 0 |
| 9 | 2 | Maugli | 2 | 0 | 0 |
| 9 | 2 | Oszkár | 3 | 0 | 0 |
| 9 | 2 | Szofi | 0 | 0 | 0 |
| 9 | 2 | Arwen | 3 | 0 | 0 |
| 9 | 2 | Dodi | 1 | 0 | 0 |
| 9 | 3 | narancspuli | 2 | 0 | 0 |
| 9 | 3 | barnapuli | 3 | 0 | 0 |
| 9 | 3 | pumimokusC | 3 | 0 | 0 |
| 9 | 3 | pumimocoN | 2 | 0 | 0 |
| 9 | 3 | collie2_Rachel | 3 | 0 | 0 |
| 9 | 3 | collie2_Rubin | 2 | 0 | 0 |
| 9 | 3 | collie3_Sultan | 3 | 0 | 0 |
| 9 | 3 | collie3_Shadow | 0 | 0 | 0 |
| 9 | 3 | csehfarkas1_fel | 0 | 0 | 0 |
| 9 | 3 | csehfarkas1_lila | 0 | 0 | 0 |
| 9 | 3 | groenisötétkék | 2 | 0 | 0 |
| 9 | 3 | groenivilkék5 | 1 | 0 | 1 |
