# Supplementary material for: Comparing the tractability of young hand-raised wolves (Canis lupus) and dogs (Canis familiaris)
Source: Sci Rep. 2020 Sep 7;10:14678. doi: 10.1038/s41598-020-71687-3 (PMC7477132; doi:10.1038/s41598-020-71687-3)

Comparing the tractability of young hand-raised wolves (*Canis lupus*) and dogs (Canis familiaris)

Dorottya Júlia Ujfalussy [2*], Zsófia Virányi [3, 4], Márta Gácsi [1, 2], Tamás Faragó [2], Ákos Pogány 2, Boróka Mária Bereczky 1, Ádám Miklósi 2, Enikő Kubinyi 2

1 MTA-ELTE Comparative Ethology Research Group
2 Department of Ethology, ELTE Eötvös Loránd University, Budapest
3 Comparative Cognition, Messerli Research Institute,
University of Veterinary Medicine, Vienna, Medical University of Vienna, University of Vienna
4 Wolf Science Center, Domestication Lab, Konrad Lorenz Institute of Ethology,
University of Veterinary Medicine Vienna, Vienna, Austria

Table 1. Mean latencies and Standard Errors in Test 2 - Calling Experiment at the tested ages (hw= hand-raised wolf, hd= hand-raised dog, md= mother-raised dog, w=weeks)

Age in weeks	hw	SE	hd	SE	md	SE
3 w	51,95	20,32	104,4	24,64		
4 w	61,63	18,61	85,66	25,95		
5 w	32,63	15,69	19,65	9,2		
6 w	15,77	4,29	15,5	5,36	23,17	4,52
7 w	34,3	15,19	11,28	4,57		
8 w	9,11	4,14	13	5,15	10,83	4,16
12 w	49,79	16,6	23,89	8,98		
16 w	29,48	11,9	8,44	2,35		
24 w	103,48	32,08	23,37	6,15		

Figure 1. Mean latencies and Standard Errors in Test 2 - Calling Experiment at the tested ages (hw= hand-raised wolf, hd= hand-raised dog, md= mother-raised dog, w=weeks)

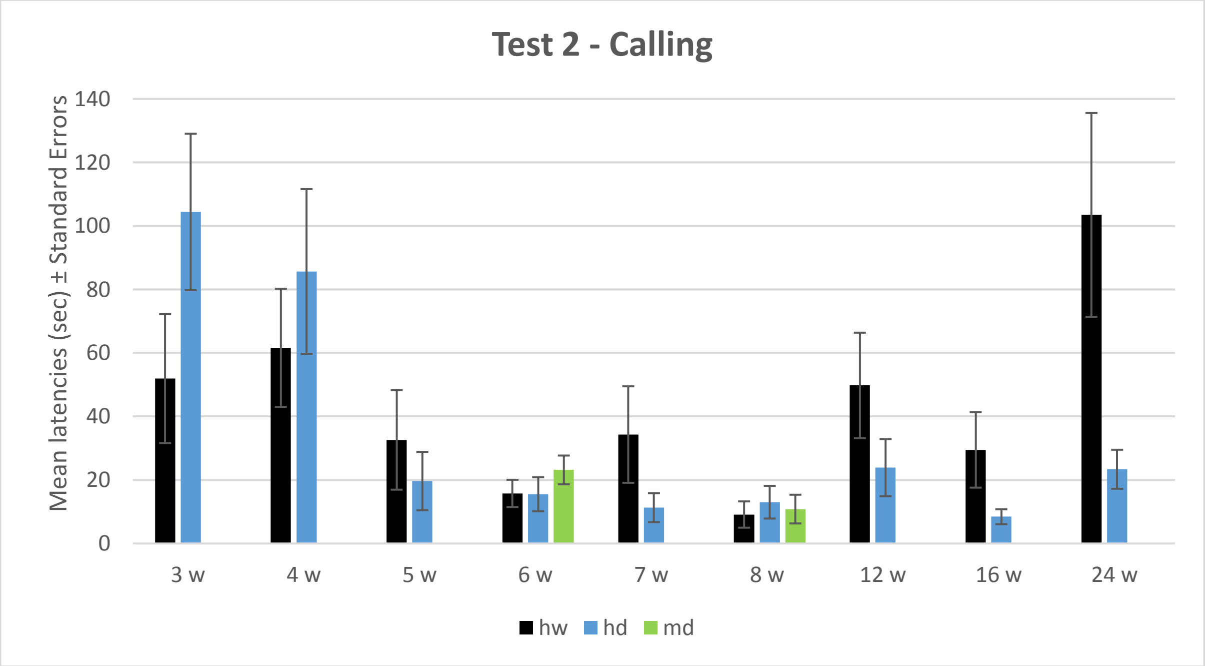

Table 2. Mean latencies and Standard Errors in Test 3 - Sitting Experiment at the tested ages (hw= hand-raised wolf, hd= hand-raised dog, md= mother-raised dog, w=weeks)

Age in weeks	hw	SE	hd	SE
7 w	148,3	41,8	53,1	11,12
9 w	90,3	29,8	119,1	17
12 w	94,8	21	118,9	20,2
16 w	114,1	21,7	58,2	19,2
24 w	83,3	15,15	64,8	10,38

Figure 2. Mean latencies and Standard Errors in Test - Sitting Experiment at the tested ages (hw= hand-raised wolf, hd= hand-raised dog, md= mother-raised dog, w=weeks)

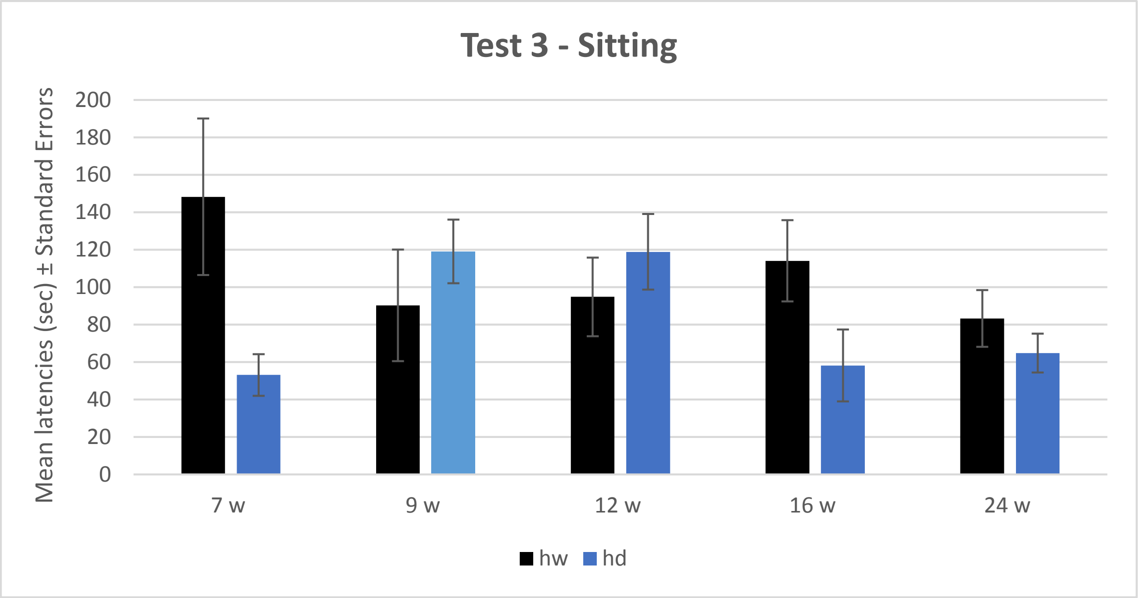

Supplement: Supplementary file 3 — Supplementary Information 3. [file 41598_2020_71687_MOESM3_ESM.pdf]
